# Mechanochemical Friedel–Crafts acylations

**DOI:** 10.3762/bjoc.15.130

**Published:** 2019-06-17

**Authors:** Mateja Đud, Anamarija Briš, Iva Jušinski, Davor Gracin, Davor Margetić

**Affiliations:** 1 Ruđer Bošković Institute, Bijenička cesta 54, HR-10002 Zagreb, Croatia

**Keywords:** ball milling, Friedel–Crafts reaction, mechanochemistry

## Abstract

Friedel–Crafts (FC) acylation reactions were exploited in the preparation of ketone-functionalized aromatics. Environmentally more friendly, solvent-free mechanochemical reaction conditions of this industrially important reaction were developed. Reaction parameters such as FC catalyst, time, ratio of reagents and milling support were studied to establish the optimal reaction conditions. The scope of the reaction was explored by employment of different aromatic hydrocarbons in conjunction with anhydrides and acylation reagents. It was shown that certain FC-reactive aromatics could be effectively functionalized by FC acylations carried out under ball-milling conditions without the presence of a solvent. The reaction mechanism was studied by in situ Raman and ex situ IR spectroscopy.

## Introduction

The Friedel–Crafts reaction (FCR) is a very powerful tool in organic chemistry for the synthesis of aromatic ketones. It is of great industrial importance and widely used in fine chemicals production [1–2]. In recent years, public awareness of the negative impact of chemical processes on the environment instigates chemists to improve processes by the reduction of waste material, energy consumption and reagents (materials). In this respect, carrying out FCR at room temperature without the use of solvents, which are usually highly toxic (halogenated hydrocarbons) will improve the eco-friendliness of the process. Until now, FCRs have been rarely applied to organic functionalizations which are carried out in solid state by mortar and pestle [3–5]. We are aware of only a few examples of FCRs employing manual grinding: reserpine acylation with AlCl_3_ [6] and acylation reaction of aromatics [7]. One of the reasons for this scarcity is the hygroscopic nature of the aluminum trichloride catalyst [8–12] when exposed to air humidity. This problem could be easily avoided by conducting the reaction in a closed vessel, by the aid of automated ball milling, which became a very effective synthetic method in recent time [13–18]. The first account on mechanochemical FC alkylation by Borchardt [19] demonstrates the utility of the mechanochemical method in the synthesis of covalent triazine frameworks. Herein, we report related results on solvent-free FC acylation reactions conducted in a ball mill, which is the continuation of our program in organic mechanosynthesis [20–24].

## Results and Discussion

**Scheme 1:**
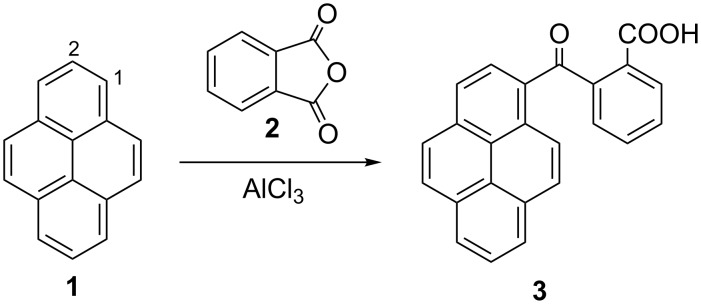
FCR of pyrene and phthalic anhydride.

Mechanochemical FCR of pyrene (**1**) and phthalic anhydride (**2**) producing 1-(*o*-carboxybenzoyl)pyrene (**3**) was selected for the optimization of reaction conditions since all reagents and catalyst are solids (Scheme 1, Table 1) [25]. In solution, this reaction is facile and the product could be obtained in quantitative yield (Table 1, entry 15). The results on optimization of reaction conditions in the ball mill indicate that FC acylation could be effectively carried out mechanochemically. The best mechanochemical reaction conditions (Table 1, entry 4): 2 h, equimolar amount of phthalic anhydride and 2.5 equivalents of AlCl_3_, afforded product **3** in high yield (79%). Identical yields were obtained by the change of reaction time to 1 h and alternative work-up procedures (Table 1, entries 1 and 4). When the catalyst amount was decreased to one equivalent, a significant decrease of yield was attained (Table 1, entry 2). Addition of various grinding additives to improve mass transfer and prevent pasting of the reaction mixture [26–28] (Table 1, entries 5–8) was detrimental to reaction yields. The addition of a small amount of solvents which was reported to facilitate several ball milling reactions (liquid assisted grinding, LAG) [29–32], also decreased yields (Table 1, entries 9 and 10). The reaction carried out in a planetary mill (Table 1, entry 11) afforded yields comparable to the MM400 vibrational mill. We have also performed screening of efficacy of various Lewis acid catalysts [33–38] (Table 1, entries 18–23), which did not lead to formation of products.

**Table 1:** Reaction of pyrene with phthalic anhydride^a^.

Entry	Conditions	Work-up^b^	Yield (%)^c^

1	1 h	B	78
2	1 h, ratio 1:1:1	A	44
3	1 h	A	76
**4**	**2 h**	**A**	**79**
5	1 h, silicagel 1 g	A	n.r.
6	1 h, dry silicagel 0.5 g	A	42
7	1 h, dry NaCl 0.5 g	A	37
8	1 h, dry Na_2_SO_4_ 0.5 g	A	43
9	1 h, LAG dry DCM	A	51
10	1 h, LAG dry THF	A	16
11	1 h, planetary mill^d^	A	79
12	1 h, teflon jar	A	71
13	3 h, reflux, dry DCM	B	94 [39]
14	1 h, reflux, dry DCM	B	98
15	1 h, reflux, dry DCM	A	83
16	1 h, rt, dry DCM	A	99
17	10 min, melt, 180 °C, dry NaCl	C [40]	n.r.^e^
18	1 h, FeCl_3_	A	n.r.
19	1 h, ZnCl_2_	A	n.r.
20	1 h, ZnI_2_	A	n.r.
21	1 h, ZnBr_2_	A	n.r.
22	1 h, CuBr_2_	A	n.r.
23	1 h, CuCl_2_	A	n.r.
24	3 h, scale-up	A	73^f^

^a^Retsch MM400 ball mill, 16 mL stainless steel vial, 1 × 12 mm stainless steel ball, 30 Hz, substrate/anhydride/AlCl_3_ ratio 1:1:2.5; ^b^Work-up A: mixture suspended in H_2_O, pH adjusted with conc. HCl, chromatography; work-up B: identical to work-up A, but recrystallisation from AcOH instead of chromatography; work-up C: suspended in aq oxalic acid, extracted with DCM, chromatography; ^c^isolated yields; ^d^Retsch planetary ball mill PM-200, 500 rpm, 25 mL stainless steel vial, 30 × 3 mm steel balls; ^e^melted in open flask; ^f^scaled up to 500 mg of pyrene.

**Scheme 2:**
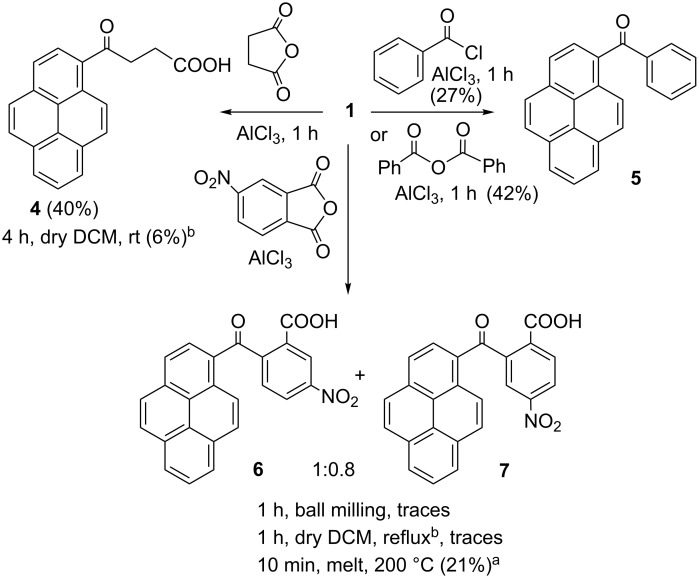
Scope of acylation reagents in FCR under mechanochemical activation conditions and comparison with other reaction conditions (isolated yields); ^a^conversion from NMR analysis; ^b^solution reaction in flask, substrate/acylation reagent/AlCl_3_ ratio is 1:1:2.5; ball-milling details are given in Table 1.

**Scheme 3:**
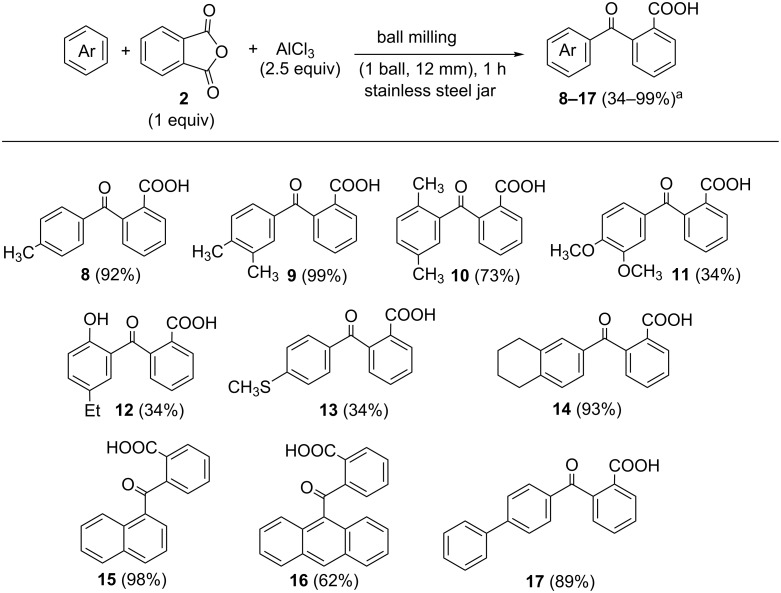
Scope of aromatic substrates in FCR under mechanochemical activation conditions. ^a^Isolated yields.

Experiments collected in Table 1 demonstrate that a FC acylation reaction could be effectively carried out under ball-milling conditions at room temperature without the use of solvent. This reaction could be easily scaled up from 94 to 500 mg of pyrene without the decrease in yield (Table 1, entry 24) [41–42]. To investigate the scope of the reaction, several acylation reagents were employed in conjunction with pyrene (Scheme 2) and a variety of aromatic substrates was subjected to FC acylation (Scheme 3).

Acylation reagents shown in Scheme 2 were less reactive in comparison to phthalic anhydride. Benzoic anhydride was used as a substitute for benzoyl chloride and the reaction proceeded in better yield. The observed disparity in reactivity might be associated with the difference in the physical state of the reagents. Furthermore, succinic anhydride poorly reacted with pyrene, but the reaction proceeds well with the more reactive biphenyl (69%, see Supporting Information File 1). Di-*tert*-butyl dicarbonate and 4-nitrobenzoyl chloride were unreactive under ball-milling conditions. Similary unreactive was 4-nitrophthalic anhydride, which only in forced conditions (by melting at 200 °C) reacted sluggishly with pyrene affording mixture of regioisomeric products **6** and **7**. The advantage of the employment of mechanochemical conditions is evidenced by solid state milling of pyrene with succinic anhydride which showed remarkably better performance than the reaction carried out in solution (40% vs 6% yield).

The screening of substrates showed disparate reactivities, ranging from quantitative to low (Scheme 3). Most rewarding are reactions of toluene, *o*-xylene, naphthalene and tetralin. Interestingly, ball milling of 4-ethylanisole provided phenol **12**, in which acylation was accompanied with the cleavage of the methoxy group [43–45]. A striking advantage of the automated ball milling over manual grinding [46] is evident in the reaction of anthracene with phthalic anhydride which gave no product by manual grinding and the yield of the toluene reaction is increased from 68% to 92%.

**Scheme 4:**
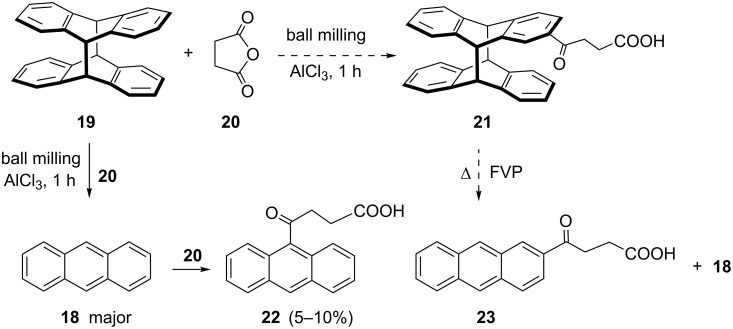
Mechanochemical regiodirected FCR of anthracene dimer and succinic anhydride.

When anthracene was subjected to a milling reaction with succinic anhydride, 9-substituted product **22** was obtained in low yield, and accompanied with a small amount of 2-acylated product **23** (Scheme 4), with same regioselectivity to that reported in the literature [47–48]. FC acylation at the 2-position of anthracene was achieved by Levy by the employment of 9,10-dihydroanthracene and subsequent oxidation to anthracene. To direct the acylation towards the 2-position, we devised the use of anthracene photodimer **19** [49] for the protection of 9,10-positions. The photodimer would act as 9,10-dihydroanthracene, and 2-acylated product should be regioselectively formed, which could be converted by thermal retrocyclization via flash vacuum pyrolysis (FVP) [50–51] to **23**. However, ball milling of **19** with **20** provided 95% conversion of **19** to anthracene, with a small amount (<5%) of **22**. This result indicates that rapid [4π – 4π] cycloreversion of **19** takes place, even in solid state ball-milling conditions at room temperature. Produced anthracene then subsequently participates in FCR. In control reaction of milling of photodimer **19** itself for 1 h was converted to anthracene in 95% yield. This [4π + 4π] cycloreversion in mechanochemical conditions is analogous to previously described dissociation of labile anthracene/C_60_ cycloadduct [52]. When the reaction of **19** with **20** was carried out in solution (DCM, overnight), 60% of dimer was converted to anthracene, and traces of FC product **22** were observed. Further attempts were made to lower the reaction temperature by cryomilling [53] (reaction vessel was cooled down by dipping into liquid nitrogen every 3–5 min, and ball milled for 30 min in total). This procedure partially suppressed cycloreversion and led to the mixture of **19** and **18** (3:2 ratio), accompanied with a small amount of **22**.

**Scheme 5:**
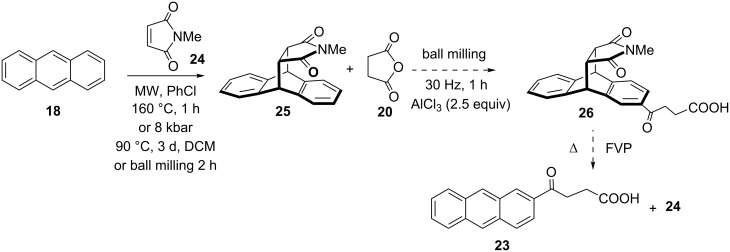
Regioselectivity direction by protection of 9,10-anthracene ring positions.

As a substitute for dianthracene **19**, thermally more stable substrate, anthracene-*N*-methyl maleimide adduct **25** [54] was prepared by Diels–Alder reaction under high pressure conditions as well as by microwave-assisted reaction and mechanochemically (Scheme 5). In this molecule, *N*-methylmaleimide could be used as protection of the 9,10-positions of anthracene and then removed by FVP. We thought that the maleimide moiety will not be affected in the FC acylation, since the precedencies exist in the literature on imide moiety withstanding the FC reaction [55–56]. However, mechanochemical reaction of **25** with succinic anhydride and 2.5 equiv of AlCl_3_ showed no reaction and the increase of the excess of catalyst to 5 equiv gave a very complex mixture.

**Scheme 6:**
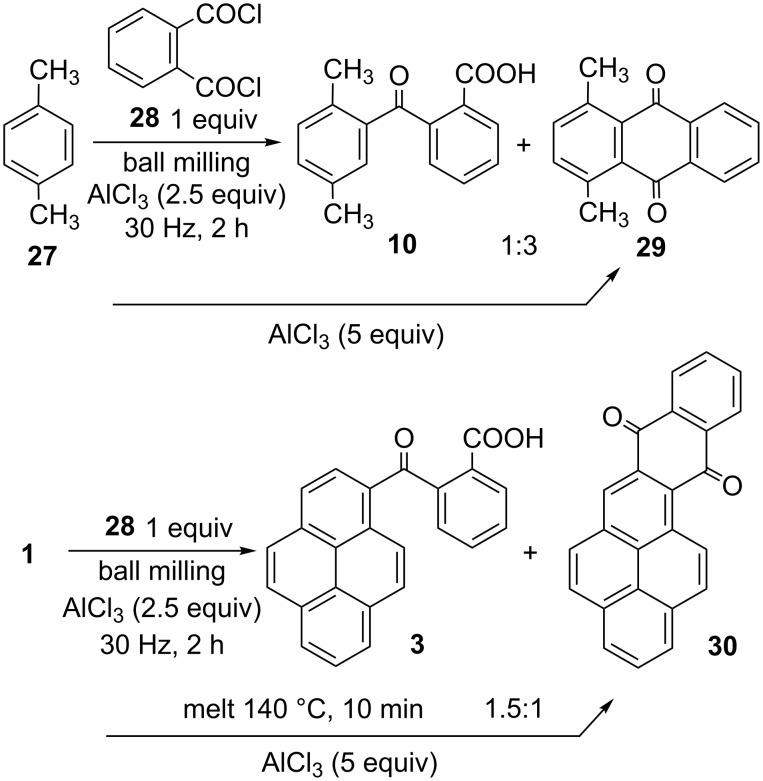
Double FCR of phthaloyl chloride and aromatics.

Phthaloyl chloride was applied in mechanochemical FCR with the goal of obtaining a double reaction leading to the anthraquinone core in a single reaction pot in solid state [57–58]. Indeed, milling of *p*-xylene, AlCl_3_ and phthaloyl chloride led to the formation of a mixture of **10** and intramolecular FC product **29** [59] in a 1:3 ratio (Scheme 6). The ratio of 1,4-dimethylanthraquinone (**29**) did not increase in the presence of 5 equivalents of AlCl_3_. Formation and ratio of these two products could be conveniently established by ^1^H NMR analysis, due to a difference in the symmetry of products: there are two methyl signals for **10** and a single methyl line at δ 2.81 ppm in the case of **29**. Pyrene and naphthalene were less reactive under the same ball milling conditions and reactions stopped at the stage of formation of product **3** and **15**. One-step preparation of quinone **30** [39], was achieved by melting reactants at 140 °C for 10 min. Under these conditions, a mixture of adducts **3** and **30** (1.5:1 ratio) was obtained. The product ratio was established by ^1^H NMR analysis of the characteristic H-10 proton signal of product **3** (peak resonance doublet at δ 9.2 ppm), which is shifted towards lower magnetic field in quinone **30** (δ 10.0 ppm), and concurrent appearance of singlet for H-3 at δ 9.1 ppm. These experiments demonstrate that quinones could be prepared by simple one-pot FC protocols in the case of reactive aromatics.

**Figure 1:**
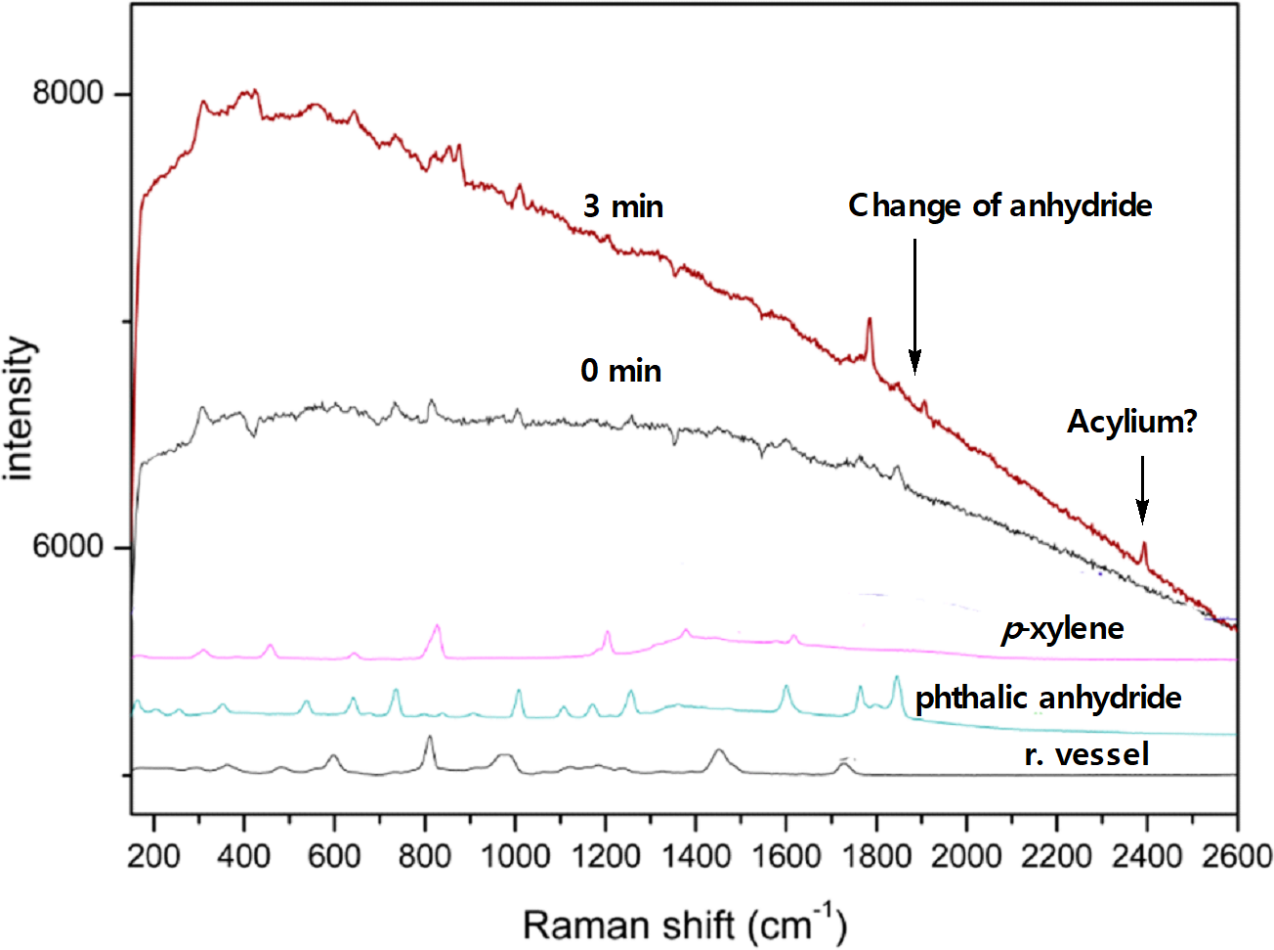
In situ Raman monitoring of reaction of phthalic anhydride with *p*-xylene.

In situ Raman spectroscopy [60] was applied to study mechanistic aspects of the solid state reaction of phthalic anhydride with *p*-xylene. Raman spectra were simulated and positions of signals for transient reactive intermediates were predicted by density functional theory method B3LYP/6-31G* (Supporting Information File 1) [61]. The stretching of the ^+^C≡O bond of the acylium ion was predicted to be at about 2300 cm^−1^. Raman spectroscopy revealed that the complexation of phthalic anhydride with AlCl_3_ is rapid, and within 3 minutes of milling all anhydride is consumed (Figure 1). After 3 minutes of milling, high fluorescence prevents further following of the reaction progress. These spectra indicate that rapid complexation of anhydride with AlCl_3_ takes place, whereas the formation of the acylium ion intermediate could not be unequivocally verified.

Similar conclusions could be drawn from ex situ IR spectroscopy [62] which indicates rapid complexation and disappearance of phthalic anhydride (Supporting Information File 1, Figures S43 and S44). A further study was carried on complexation of phthalic anhydride with AlCl_3_ (Supporting Information File 1, Figure S45). Although there are weak signals at 2300 and 3050 cm^−1^ which could be associated with the acylium ion and the intermediate cation, the raise of intensities of these signals over the time is quite unlikely to come from reactive species (time needed to transfer sample from ball mill to IR spectrophotometer and acquire spectra are within several minutes, which could be detrimental to reactive species to survive in the open air). These signals are not visible after the standard acidic work-up and further study would require the use of in situ IR spectroscopy in solution [63].

## Conclusion

In conclusion, the experimental results demonstrate that Friedel–Crafts acylations could be effectively carried out under solid state ball-milling conditions. The reaction takes place by the initial complexation of the carbonyl group of the acylation reagent with aluminium trichloride.

## Supporting Information

File 1 Details of experimental procedures, spectroscopic characterization data of compounds and computational procedures.
