# Supplementary material for: Mechanochemical Friedel–Crafts acylations
Source: Beilstein J Org Chem. 2019 Jun 17;15:1313–20. doi: 10.3762/bjoc.15.130 (PMC6604704; doi:10.3762/bjoc.15.130)
Supplement: File 1 — Details of experimental procedures, spectroscopic characterization data of compounds and computational procedures. [file Beilstein_J_Org_Chem-15-1313-s001.pdf]

BEILSTEIN JOURNAL OF ORGANIC CHEMISTRY

Supporting Information

for

Mechanochemical Friedel–Crafts acylations

Mateja Đud, Anamarija Briš, Iva Jušinski, Davor Gracin and Davor Margetić

Beilstein J. Org. Chem. **2019,** *15,* 1313–1320. doi:10.3762/bjoc.15.130

Details of experimental procedures, spectroscopic characterization data of compounds and computational procedures

License and Terms: This is a supporting information file under the terms of the Creative Commons Attribution License (http://creativecommons.org/licenses/by/4.0). Please note that the reuse, redistribution and reproduction in particular requires that the authors and source are credited.
The license is subject to the *Beilstein Journal of Organic Chemistry* terms and conditions: (https://www.beilstein-journals.org/bjoc)

General

Commercially available reagents were supplied from the following chemical manufacturers: Sigma-Aldrich, Alfa Aesar or Acros Organics and used without further purification, unless otherwise indicated. Solvents were obtained from Kemika, Sigma-Aldrich or VWR Chemicals. For the purpose of carrying out the reaction under dry conditions, the solvents were dried using standard methods.

Chromatography: Thin layer chromatography on alumina coated silica gel plates (silica gel 60 F_{254}, Merck) was used to monitor the course of the reaction. The analysis was performed using UV lamps of wavelengths 254 and 365 nm. Purification and separation of the synthesized products were performed by column chromatography using silica gel (Silica gel 60, 0.063-0.200 mm, Merck). Elution solvents and their ratios are specified for each individual synthesis.

Mehanochemical reactions: Grinding reactions were performed on a vibrational mill Retsch MM400 at a frequency of 30 Hz using 16 mL stainless steel vessels and one 12 mm steel ball. Retsch planetary ball mill PM-200, 500 rpm, using 25 mL stainless steel vessels and 30x3 mm steel balls.

High pressure reactions: High-pressure experiments were performed using a high pressure piston-cylinder apparatus (Unipress, Polish Academy of Sciences) and pentane as piezotransmitting liquid in Teflon cells (V=1.5 mL).

Microwave reactions: Reactions were conducted in a CEM Discover LabmateTH/ExplorerPLS single-mode microwave reactor using the closed reaction vessel technique with power=125 W.

Nuclear magnetic resonance (NMR): The compounds were identified by one-dimensional ^{1}H and ^{13}C spectroscopy, using Bruker Avance 300 MHz and Bruker Avance 600 MHz spectrometers. Commercially available deuterated solvents were used for the recording. Chemical shifts (δ) are expressed in ppm according to tetramethylsilane (TMS) as internal standard, and coupling constants (*J*) are expressed in Hertz (Hz). The following abbreviations were used to denote multiplicity in ^{1}H spectra: s, singlet; d, doublet; dd, doublet of doublets; t, triplet; m, multiplet; brs, broad signal.

Infrared spectroscopy: Infrared spectra (FTIR-ATR) were recorded using Fourier Transform - Infrared Attenuated Total Reflection PerkinElmer UATR Two Spectrometer, ranging from 400 cm^{-1} to 4000 cm^{-1}.

Raman in situ spectroscopy: Retsch MM300 ball mill was operated at 30 Hz to conduct the milling reactions in poly(methyl methacrylate) (PMMA) milling vessels (V = 14 mL) with two 7 mm diameter stainless steel balls. A portable Raman spectroscopy system was assembled from the PD-LD LS2 laser source (λ = 784 nm) and Maya spectrometer (Ocean Optics). The focus of the Raman probe was adjusted at a distance of ≈8 mm from the Raman probe to be inside the vessel.

General procedure for mechanochemical Friedel–Crafts acylations

Method A. A mixture of aromatic substrate (0.46 mmol), phthalic anhydride (1 equiv) and $AlCl_3$ (2.5 equiv.) was placed in a 16 mL stainless steel jar, together with one stainless steel 12 mm ball in Retsch MM400 ball mill. The mixture was ball milled for 1 hour at 30 Hz. The reaction mixture was suspended in water, pH adjusted with conc. HCl. The precipitate was collected by filtration and subjected to column chromatography on silicagel using dichloromethane to remove unreacted aromatics, then product was eluted with DCM/10% methanol.

Caution: Aluminium trichloride dust is very irritant and corrosive to all tissues and reacts violently with water. It readily undergoes violent chemical changes at elevated temperatures and pressures. Prior to its handling, material safety data sheet (MSDS) should be consulted. The necessary precautionary measures are essential for the handling of the material. Wear appropriate eye protection (goggles, face shield) to prevent eye contact, gloves to prevent skin contact and perform all handling in well-ventilated fumehood to prevent inhalation.[1]

1-(*o*-Carboxybenzoyl)pyrene (**3**) [2]

^{1}H NMR ($CDCl_3$) δ 9.22 (d, *J*=9.8 Hz, 1H, H-10), 8.27-8.20 (m, 3H), 8.15 (d, *J*=8.7 Hz, 1H), 8.09-7.94 (m, 3H), 7.86-7.79 (m, 2H), 7.68-7.60 (m, 1H), 7.57-7.49 (m, 2H).
^{1}H NMR (DMSO-d_6) δ 13.06 (brs, 1H, COOH), 9.17 (d, *J*=9.1 Hz, 1H, H-10), 8.44 (d, *J*=7.7 Hz, 2H, H-6, H-8), 8.43 (d, *J*=9.1 Hz, 1H, H-9), 8.36 (d, *J*=9.1 Hz, 1H, H-5), 8.27-8.16 (m, 3H, H-3, H-4, H-7), 7.99 (dd, *J*=7.5, 1.6 Hz, 1H, H-6'), 7.84 (d, *J*=8.1 Hz, 1H, H-2), 7.82-7.69 (m, 2H, H-4′, H-5′), 7.64 (dd, *J*=7.3, 1.4 Hz, 1H, H-3′).
FTIR-ATR ν_{max}/cm^{-1}: 3038, 2921, 2620, 1716 (C=O).

4-Oxo-4-(pyren-1-yl)butanoic acid (**4**) [3]

^{1}H NMR (DMSO-d_6) δ 8.75 (d, *J*=9.7 Hz, 1H), 8.57 (d, *J*=8.4 Hz, 1H), 8.42-8.22 (m, 6H), 8.14 (t, *J*=7.6 Hz, 1H), 3.44 (t, *J*=6.0 Hz, 2H, CH_2), 2.69 (t, *J*=6.0 Hz, 2H, CH_2).
FTIR-ATR ν_{max}/cm^{-1}: 3041, 2912, 2671, 1692, 1664 (C=O).

1-Benzoylpyrene (**5**) [4]

^{1}H NMR ($CDCl_3$) δ 8.35 (d, *J*=8.7 Hz, 1H), 8.22-8.10 (m, 4H), 8.09-7.98 (m, 4H), 7.90-7.85 (m, 2H), 7.62-7.55 (m, 1H), 7.47-7.41 (m, 2H).
FTIR-ATR ν_{max}/cm^{-1}: 3043, 2248, 1922, 1805, 1651 (C=O).

4-Methyl-1-(*o*-carboxybenzoyl)benzene (**8**) [5]

^{1}H NMR (DMSO-d_6) δ 7.95-7.89 (m, 1H), 7.49-7.41 (m, 2H), 7.44 (d, *J*=8.4 Hz, 2H), 7.20 (d, *J*=8.4 Hz, 2H), 7.13-7.08 (m, 1H), 3.40 (brs, 1H, OH), 2.33 (s, 3H, CH_3).
FTIR-ATR ν_{max}/cm^{-1}: 3058, 2963, 1655 (C=O).

3,4-Dimethyl-1-(*o*-carboxybenzoyl)benzene (**9**) [6]

^{1}H NMR (DMSO-d_6) δ 7.94-7.89 (m, 1H), 7.49-7.44 (m, 2H), 7.39-7.36 (m, 1H), 7.25 (d, *J*=7.6 Hz, 1H), 7.15 (d, *J*=7.6 Hz, 1H), 7.13-7.10 (m, 1H), 3.37 (brs, 1H, OH), 2.24 (s, 3H, CH_3), 2.20 (s, 3H, CH_3).
FTIR-ATR ν_{max}/cm^{-1}: 3054, 1710 (C=O).

1,4-Dimethyl-2-(*o*-carboxybenzoyl)benzene (**10**) [7]

^{1}H NMR ($CDCl_3$) δ 8.01 (d, *J*=7.8 Hz, 1H), 7.68-7.61 (m, 1H), 7.59-7.53 (m, 1H), 7.42 (d, *J*=7.2 Hz, 1H), 7.16 (s, 2H), 6.99 (s, 1H), 2.55 (s, 3H, CH_3), 2.21 (s, 3H, CH_3).
FTIR-ATR ν_{max}/cm^{-1}: 3019, 2925, 2671, 2546, 1674 (C=O).

3,4-Dimethoxy-1-(*o*-carboxybenzoyl)benzene (**11**) [8,9]

^{1}H NMR ($CDCl_3$) δ 8.11-8.04 (m, 1H), 7.67-7.52 (m, 3H), 7.39-7.34 (m, 1H), 7.06 (d, *J*=8.5 Hz, 1H), 6.77 (d, *J*=8.5 Hz, 1H), 3.92 (s, 3H, OCH_3), 3.91 (s, 3H, OCH_3)
^{1}H NMR (DMSO-d_6) δ7.95 (d, *J*=7.5 Hz, 1H), 7.72-7.57 (m, 2H), 7.38 (s, 1H), 7.35 (d, *J*=7.5 Hz, 1H), 7.00-6.92 (m, 2H), 3.79 (s, 3H, OCH_3), 3.78 (s, 3H, OCH_3).
^{13}C NMR (DMSO-d_6) δ 195.5 (C=O), 167.5 (C=O), 153.5, 149.2, 141.9, 132.5, 130.9, 130.4, 130.1, 129.9, 127.9, 125.1, 111.2, 110.6, 56.2 (OCH_3).55.9 (OCH_3).
FTIR-ATR ν_{max}/cm^{-1}: 3077, 2935, 2847, 1715 (C=O).

5-Ethyl-2-hydroxy-1-(*o*-carboxybenzoyl)benzene (**12**) [10]

^{1}H NMR ($CDCl_3$) δ 11.60 (brs, 1H, OH), 9.47 (brs, 1H, OH), 8.03 (brs, 1H), 7.62 (brs, 1H), 7.52 (brs, 1H), 7.31 (brs, 1H), 7.26 (brs, 1H), 6.92 (d, *J*=8.1 Hz, 1H), 6.83 (s, 1H), 2.38 (q, *J*=7.5 Hz, 2H, CH_2), 1.02 (t, *J*=7.5 Hz, 3H, CH_3).

^{13}C NMR ($CDCl_3$) δ 202.8 (C=O), 170.9 (C=O), 160.6, 140.5, 136.6, 134.5, 132.7, 131.2, 131.1, 129.7, 127.4, 119.8, 118.1, 27.7 (CH_2), 15.5 (CH_3).
^{1}H NMR (DMSO-d_6) δ 12.23 (brs, 1H, COOH), 7.99 (d, *J*=7.1 Hz, 1H), 7.73-7.59 (m, 2H), 7.44-7.34 (m, 2H), 6.93 (dd, *J*=6.0, 3.0 Hz, 2H), 2.42 (q, *J*=7.1 Hz, 2H, CH_2), 1.02 (t, *J*=7.1 Hz, 3H, CH_3).
^{13}C NMR (DMSO-d_6) δ 164.7 (C=O), 158.9 (C=O), 140.4, 135.5, 133.9, 131.7, 131.2, 130.4, 129.6 (2C), 126.8, 120.7, 117.6, 29.6 (CH_2), 15.6 (CH_3). (1C unaccounted)
FTIR-ATR ν_{max}/cm^{-1}: 3060, 2965, 2931, 2872, 1694 (C=O).

4-Methylthio-1-(*o*-carboxybenzoyl)benzene (**13**) [11]

^{1}H NMR ($CDCl_3$) δ 8.07 (d, *J*=7.6 Hz, 1H), 7.69-7.59 (m, 2H), 7.62 (d, *J*=8.6 Hz, 2H), 7.59-7.52 (m, 1H), 7.35 (d, *J*=6.9 Hz, 1H), 7.21 (d, *J*=8.6 Hz, 1H), 2.49 (s, 3H, SCH_3)
^{13}C NMR ($CDCl_3$) δ 169.6 (C=O), 146.2 (C=O), 133.4, 133.1, 130.9, 130.0, 129.8, 129.5, 128.1, 127.8, 127.6, 124.9, 14.7 (SCH_3)
^{1}H NMR (DMSO-d_6) δ 12.95 (brs, 1H, COOH), 7.99 (dd, *J*=7.6, 1.2 Hz, 1H), 7.75-7.61 (m, 2H), 7.54 (d, *J*=8.5 Hz, 2H), 7.40 (dd, *J*=7.2, 1.2 Hz, 1H), 7.34 (d, *J*=8.5 Hz, 1H), 2.52 (s, 3H, SCH_3)
^{13}C NMR (DMSO-d_6) δ 195.4 (C=O), 166.9 (C=O), 145.4, 141.4, 133.2, 132.3, 129.9, 129.7, 129.6, 129.3, 127.3, 124.8, 33.1 (SCH_3).
FTIR-ATR ν_{max}/cm^{-1}: 3060, 2921, 2654, 2545, 1721 (C=O).

5-(*o*-Carboxybenzoyl)-1,2,3,4-tetrahydronaphthalene (**14**) [12]

^{1}H NMR ($CDCl_3$) δ 7.70-7.63 (m, 1H), 7.35-7.31 (m, 1H), 7.29-7.21 (m, 2H), 7.14-7.01 (m, 1H), 7.04 (d, *J*=7.2 Hz, 1H), 6.78 (d, *J*=7.2 Hz, 1H), 3.62 (brs, 1H, OH), 2.65-2.59 (m, 2H), 2.54-2.49 (m, 2H), 1.69-1.59 (m, 4H).
^{1}H NMR (DMSO-d_6) δ 7.94-7.88 (m, 1H), 7.48-7.41 (m, 2H), 7.28-7.22 (m, 2H), 7.13-7.08 (m, 1H), 7.06 (d, *J*=8.1 Hz, 1H), 3.44 (brs, 1H, OH), 2.76-2.62 (m, 4H, H-5, H-8, CH_2), 1.76-1.68 (m, 4H, H-6, H-7, CH_2).
FTIR-ATR ν_{max}/cm^{-1}: 2929, 2854, 1660 (C=O).

1-(*o*-Carboxybenzoyl)naphthalene (**15**) [13,14]

^{1}H NMR ($CDCl_3$) δ 8.98 (d, *J*=9.5 Hz, 1H, H-8), 8.04 (dd, *J*=7.4, 1.1 Hz, 1H), 7.98 (d, *J*=8.1 Hz, 1H), 7.89 (d, *J*=8.6 Hz, 1H), 7.72-7.60 (m, 3H), 7.59-7.53 (m, 2H), 7.44-7.32 (m, 2H), 1.98 (brs, 1H).
FTIR-ATR ν_{max}/cm^{-1}: 3057, 1659 (C=O).

2-(9-Anthroyl)benzoic acid (**16**) [15]

^{1}H NMR (DMSO-d_6) δ 8.87 (s, 1H, H-10), 8.20 (d, *J*=8.3 Hz, 2H), 8.02 (d, *J*=8.3 Hz, 2H), 7.72 (d, *J*=7.8 Hz, 1H), 7.60 (t, *J*=6.8 Hz, 1H), 7.56 (t, *J*=6.8 Hz, 2H), 7.51 (t, *J*=8.3 Hz, 2H), 7.25 (t, *J*=7.3 Hz, 1H), 6.92 (d, *J*=6.8 Hz, 1H), 3.3 (brs, 1H).
FTIR-ATR ν_{max}/cm^{-1}: 3052, 2921, 2657, 2540, 1699 (C=O)

4-Phenyl-1-(*o*-carboxybenzoyl)benzene (**17**) [16]

^{1}H NMR (DMSO-d_6) δ 13.25 (brs, 1H, OH), 8.02 (d, *J*=7.5 Hz, 1H), 7.82-7.65 (m, 8H), 7.53-7.40 (m, 4H).
FTIR-ATR ν_{max}/cm^{-1}: 3053, 3033, 2836, 2679, 2554, 1690 (C=O).

1,4-Dimethylanthraquinone (**29**) [17]

^{1}H NMR ($CDCl_3$) δ 8.22 (dd, *J*=5.8, 3.3 Hz, 2H), 7.76 (dd, *J*=5.8, 3.3 Hz, 2H), 7.44 (s, 2H), 2.81 (s, 6H, CH_3). (spectroscopic data were obtained from crude mixture)

3-(4-Phenylbenzoyl)propionic acid (Fenbufen™) (**17B**) [18,19]

^{1}H NMR (DMSO-d_6) δ 12.18 (brs, 1H, OH), 8.06 (d, *J*=8.7 Hz, 2H), 7.83 (d, *J*=8.7 Hz, 2H), 7.75 (d, *J*=7.9 Hz, 2H), 7.51 (t, *J*=7.2 Hz, 2H), 7.42 (d, *J*=7.2 Hz, 1H), 3.28 (t, *J*=6.2 Hz, 2H), 2.60 (t, *J*=6.2 Hz, 2H).

Anthracene photodimer (**19**) [20]

A solution of anthracene 100 mg (0.56 mmol) in dry benzene (15 mL) was placed in a round-bottom Pyrex flask which was fitted with a reflux condenser. It was irradiated with a 150-W lamp placed close to flask and the heat from the lamp brought up the solution to gently reflux. After 24 h, the irradiation was stopped and the solution was cooled to room temperature. The colorless crystals that precipitated were isolated via vacuum filtration, washed with a few mL of ice-cold benzene, and dried under vacuum to afford 53 mg (53% yield) of photodimer **19**.

^{1}H NMR ($CDCl_3$) δ 6.92 (dd, *J*=5.5, 3.2 Hz, 8H), 6.81 (dd, *J*=5.5, 3.2 Hz, 8H), 4.54 (s, 4H).

13-Methyl-10,11-dihydro-9*H*-9,10-[3,4] epipyrroloanthracene-12,14(13*H*,15*H*)-dione (**25**) [21]

Method 1.[22] A solution of anthracene (50 mg, 0.281 mmol) and *N*-methylmaleimide (50 mg, 0.450 mmol) in dichloromethane (1.5 mL) was pressurized at 8 kbar and 90 °C for 3 days. Solvent and unreacted reagents were removed in high vacuo to afford cycloadduct **25** as colorless solid (68 mg, 84%).
Method 2.[23] A solution of anthracene (50 mg, 0.281 mmol) and *N*-methylmaleimide (50 mg, 0.450 mmol) in chlorobenzene (1 mL) was subjected to microwave irradiation at 160 °C for 1 hour.

Solvent and unreacted reagents were removed in high vacuo to afford cycloadduct **25** as colorless solid (61 mg, 75%).

Method 3. A mixture of anthracene (50 mg, 0.281 mmol) and *N*-methylmaleimide (31 mg, 0.281 mmol) was subjected to neat grinding by ball milling (stainless steel 12 mm ball, 30 Hz) for 2 hours. Solid was collecetd by scraping off the reaction vessel and ball to afford cycloadduct **25** as colorless solid (76 mg, 94%).

^{1}H NMR ($CDCl_3$) δ 7.37 (dd, *J*=5.4, 3.3 Hz, 2H), 7.26 (dd, *J*=5.4, 3.3 Hz, 2H), 7.17 (dd, *J*=5.6, 3.2 Hz, 2H), 7.11 (dd, *J*=5.6, 3.2 Hz, 2H), 4.78 (s, 2H), 3.22 (s, 2H), 2.58 (s, 3H, NCH_3).

NMR spectra

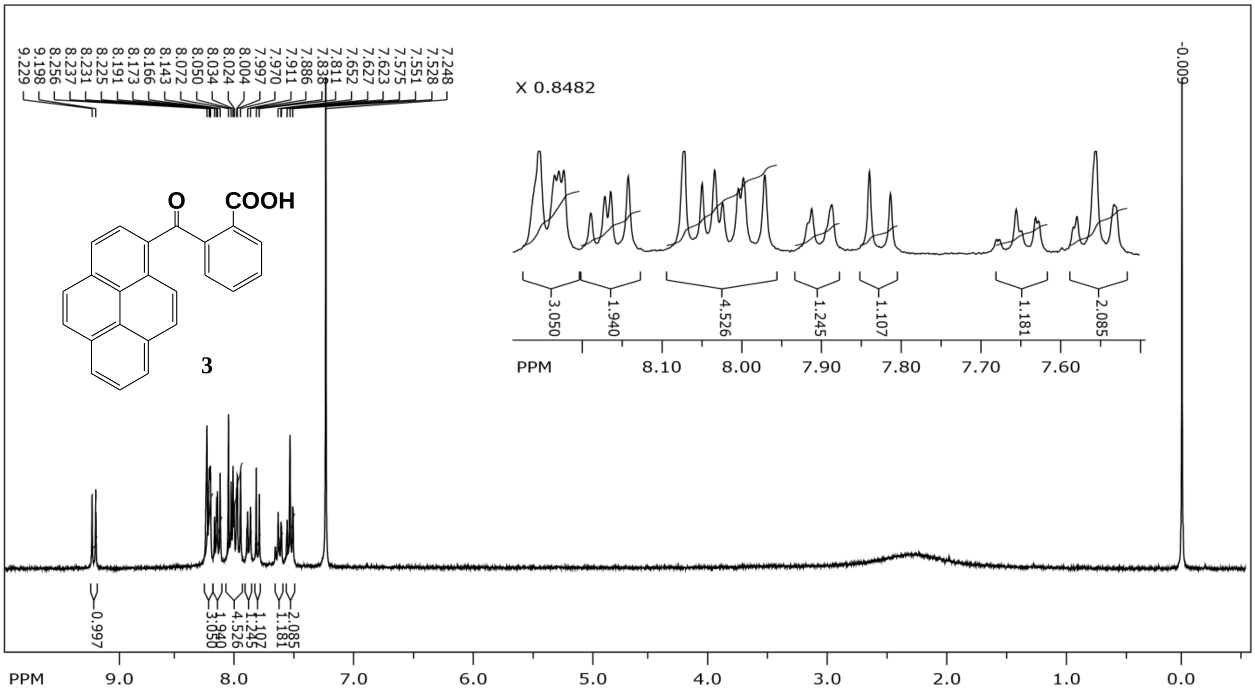

Figure S1. ^{1}H NMR (300 MHz, $CDCl_3$) spectrum of **3**

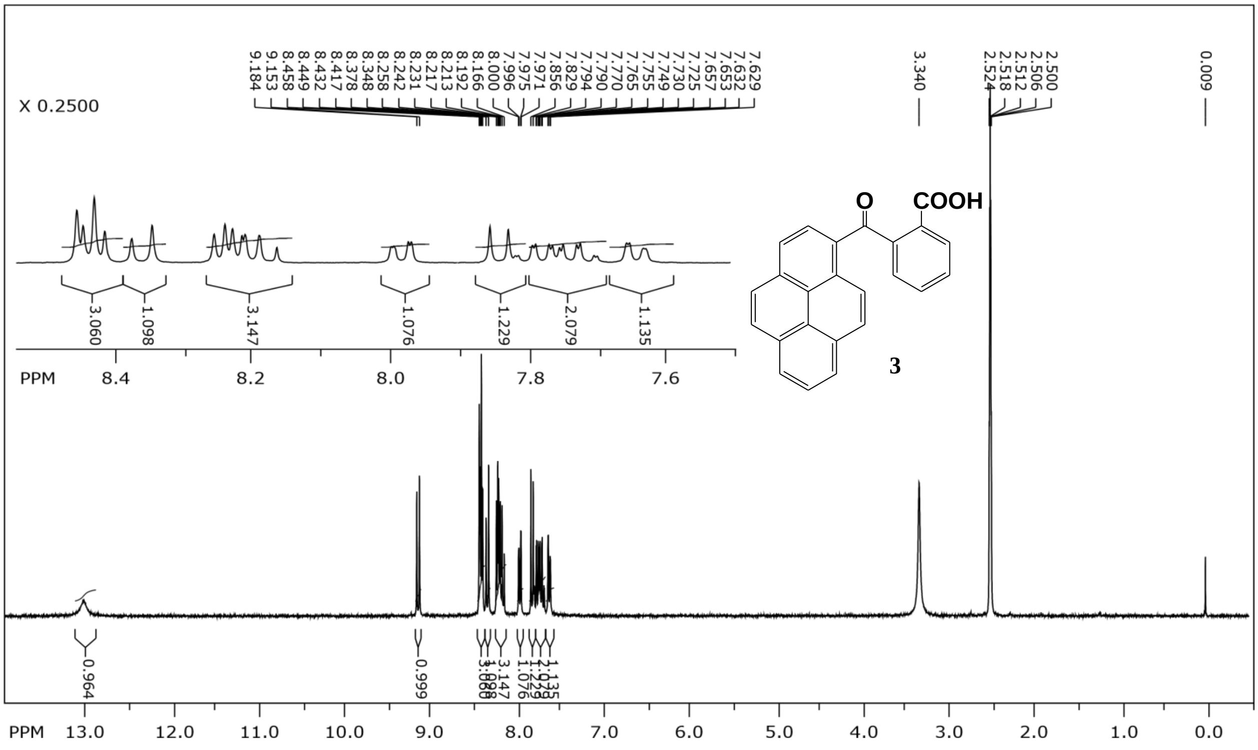

Figure S2. ^{1}H NMR (300 MHz, DMSO-d_6) spectrum of **3**

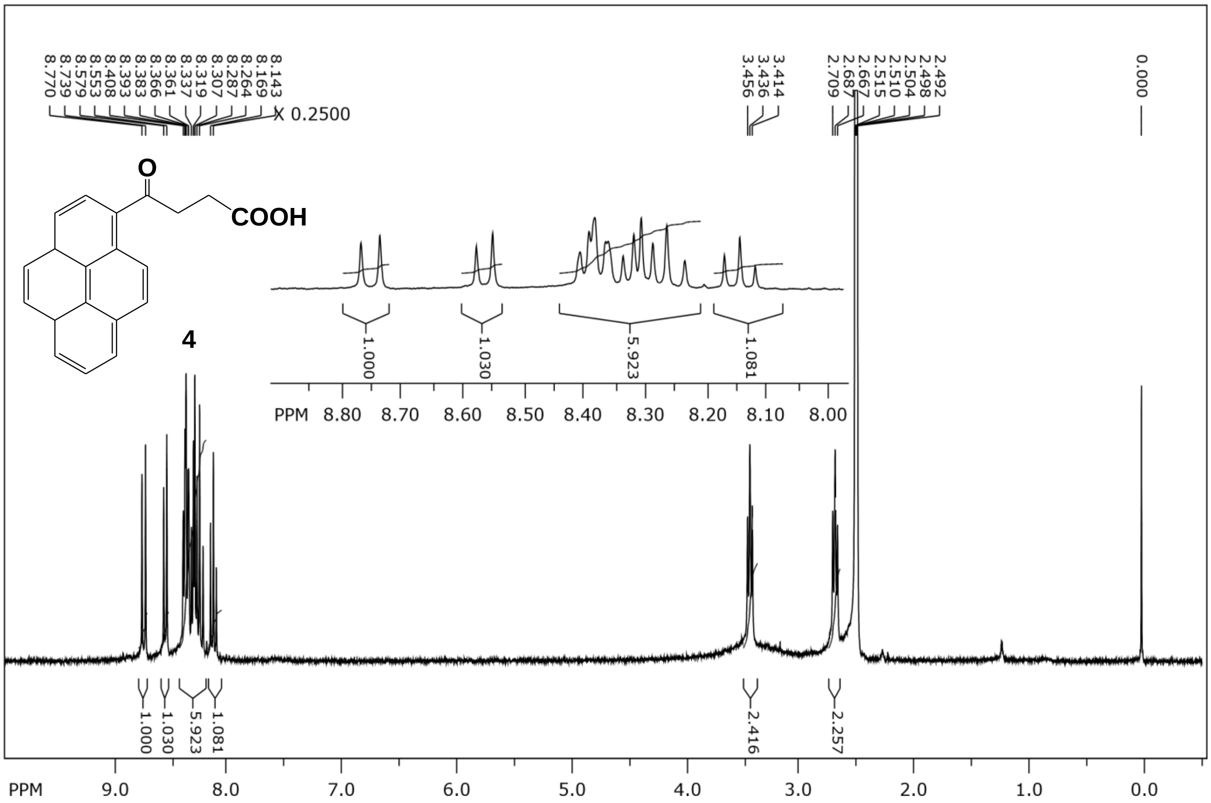

Figure S3. ^{1}H NMR (300 MHz, DMSO-d_6) spectrum of **4**

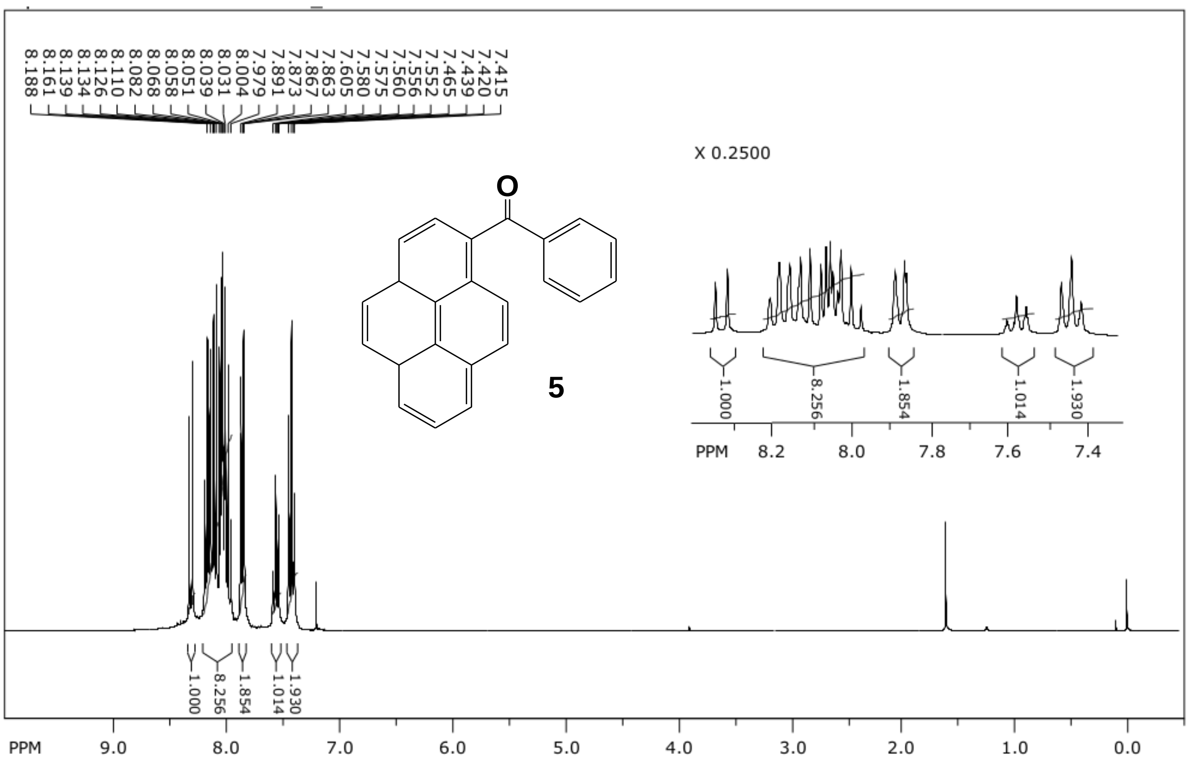

Figure S4. ^{1}H NMR (300 MHz, $CDCl_3$) spectrum of **5**

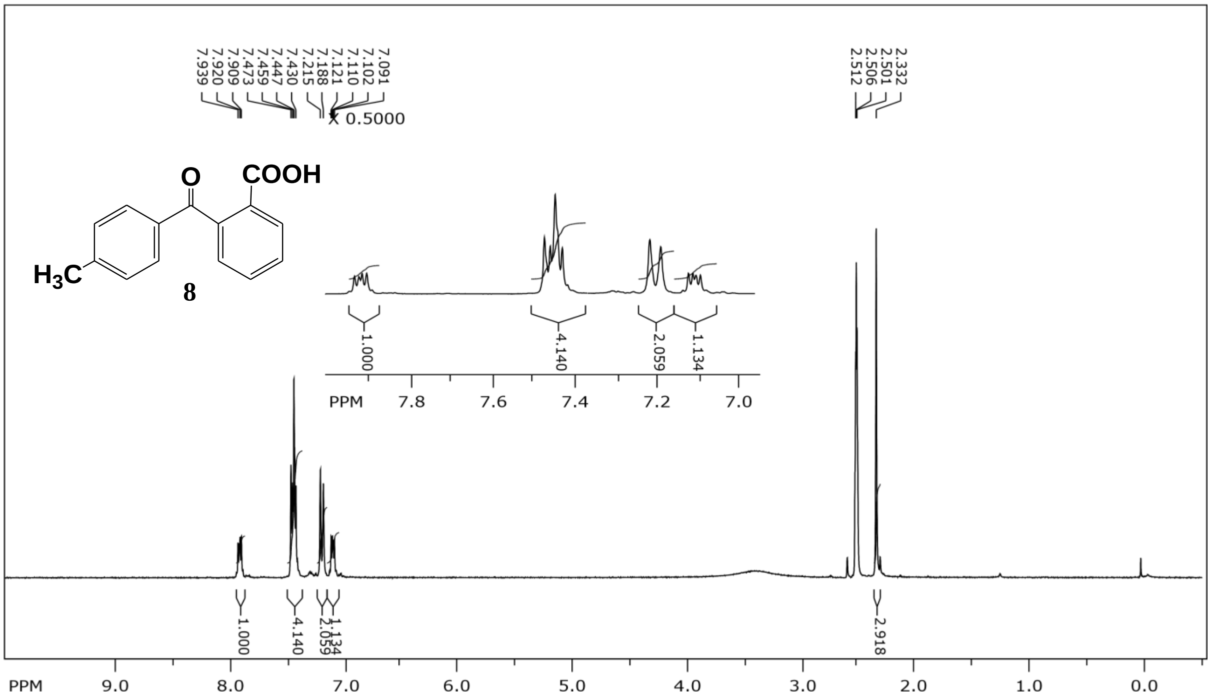

Figure S5. ^{1}H NMR (300 MHz, DMSO-d_6) spectrum of **8**

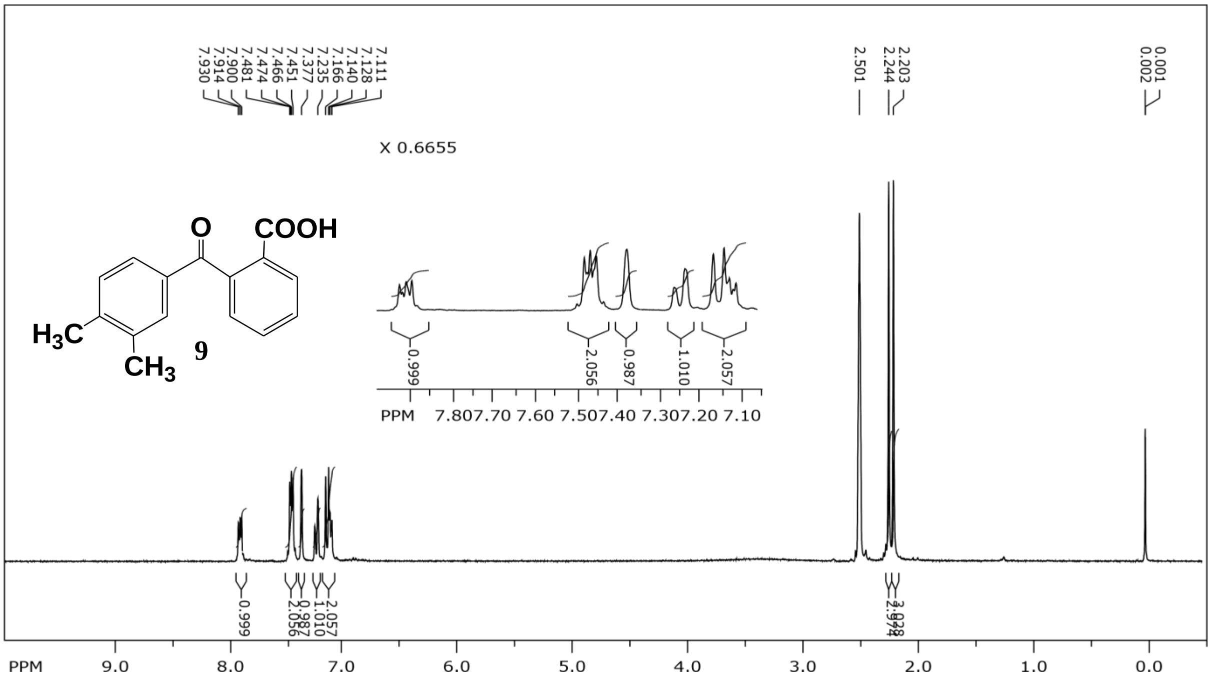

Figure S6. ^{1}H NMR (300 MHz, DMSO-d_6) spectrum of **9**

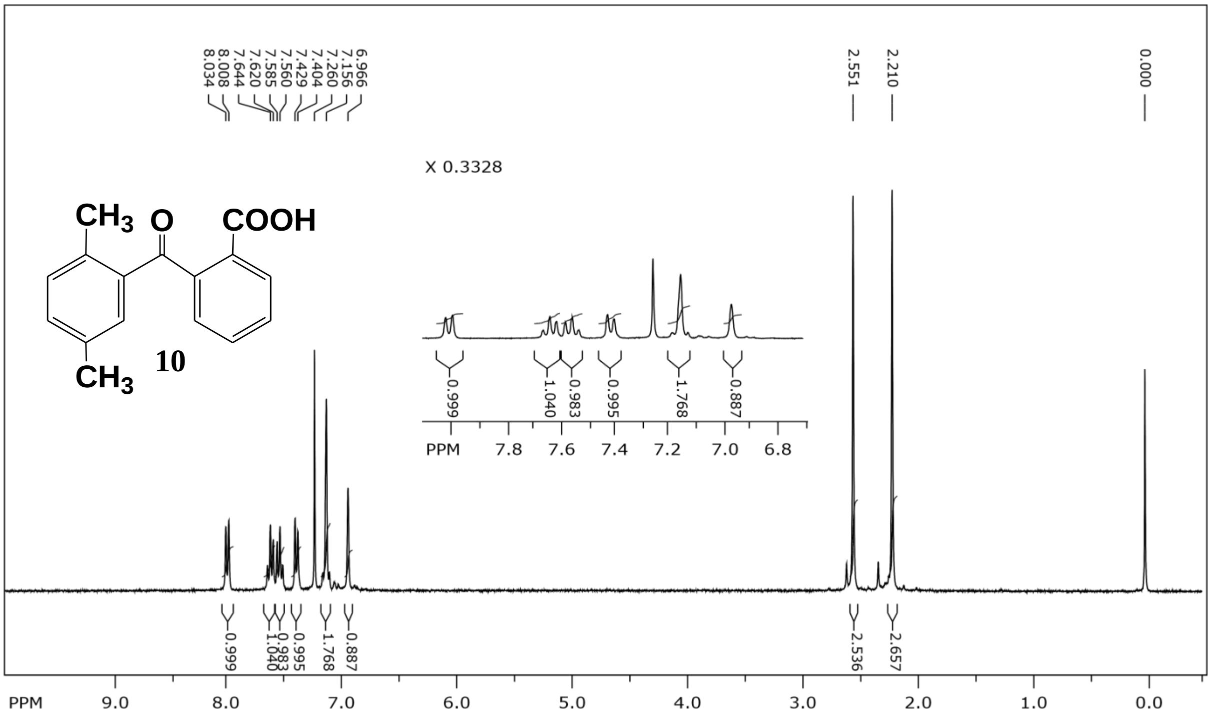

Figure S7. ^{1}H NMR (300 MHz, $CDCl_3$) spectrum of **10**

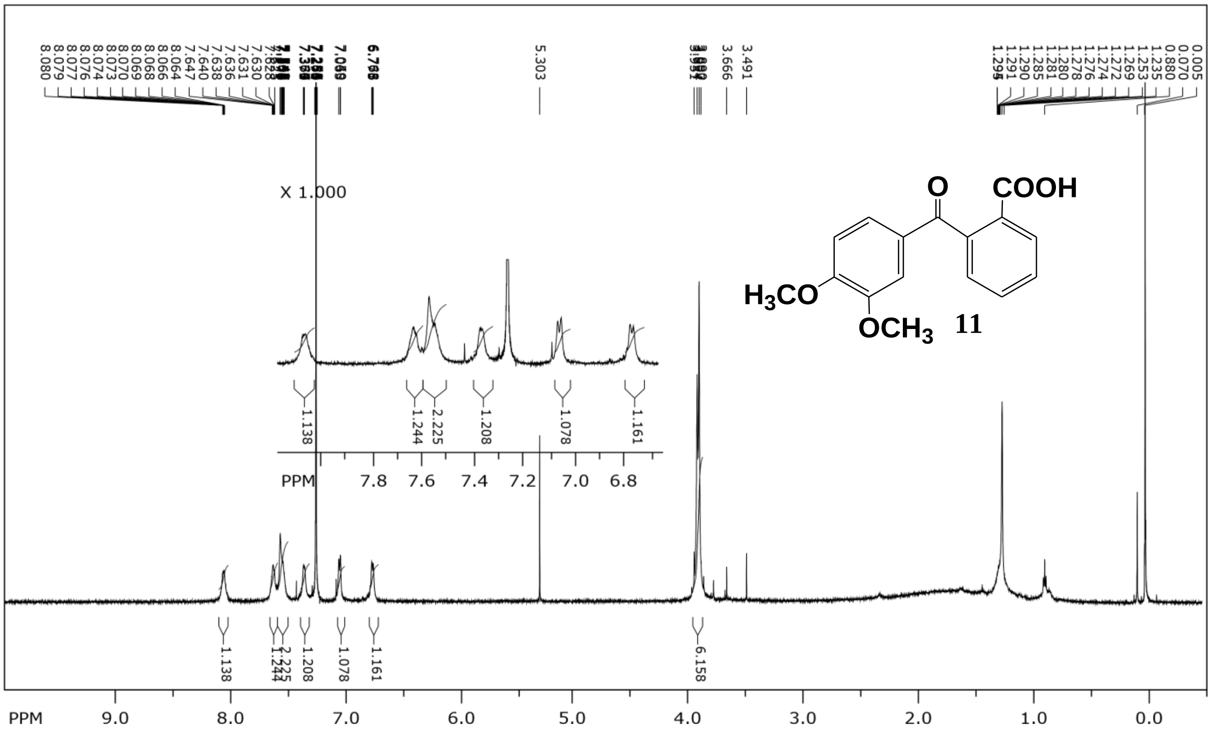

Figure S8. ^{1}H NMR (300 MHz, $CDCl_3$) spectrum of **11**

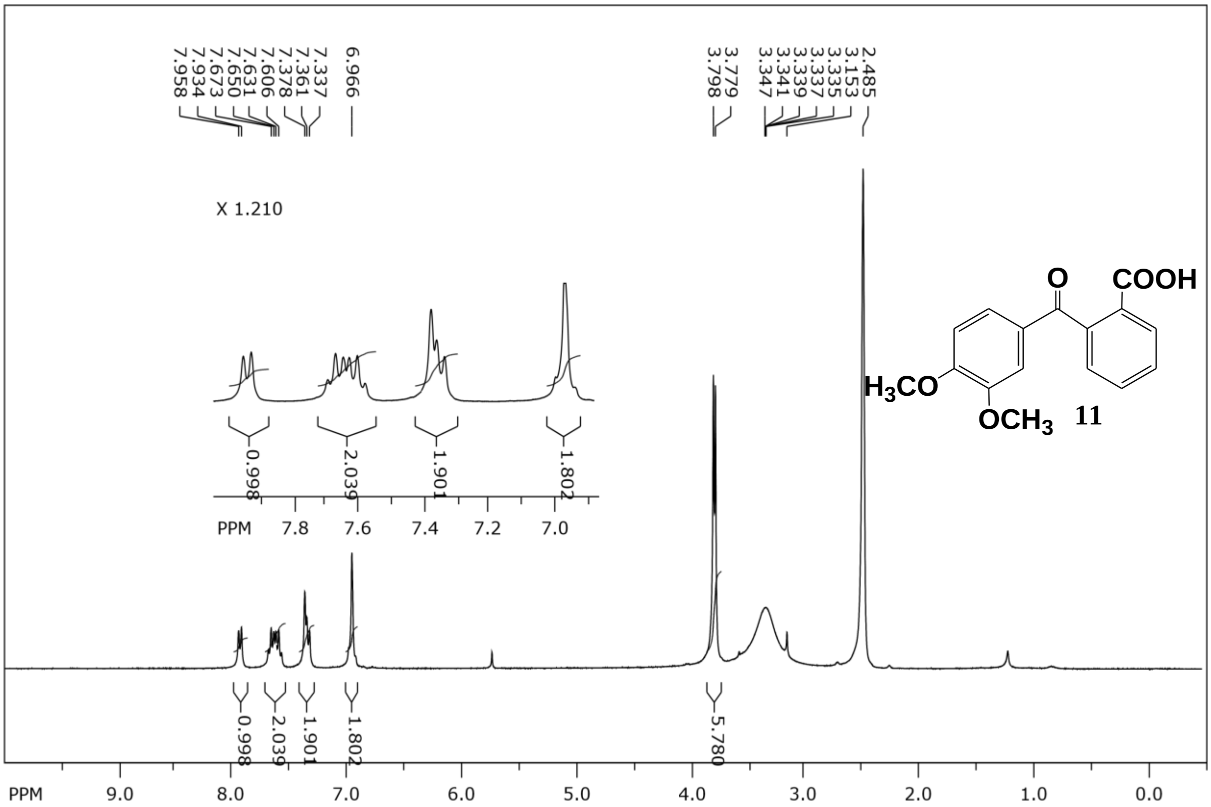

Figure S9. ^{1}H NMR (300 MHz, DMSO-d_6) spectrum of **11**

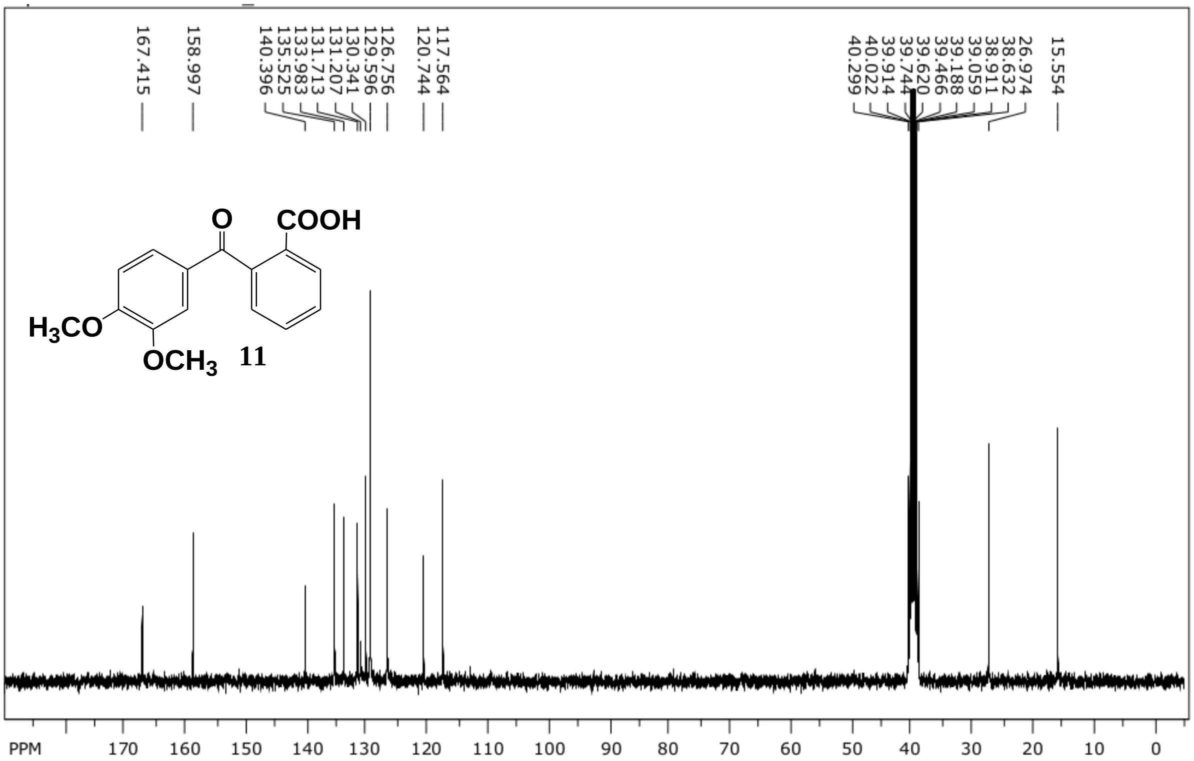

Figure S10. ^{13}C NMR (300 MHz, DMSO-d_6) spectrum of **11**

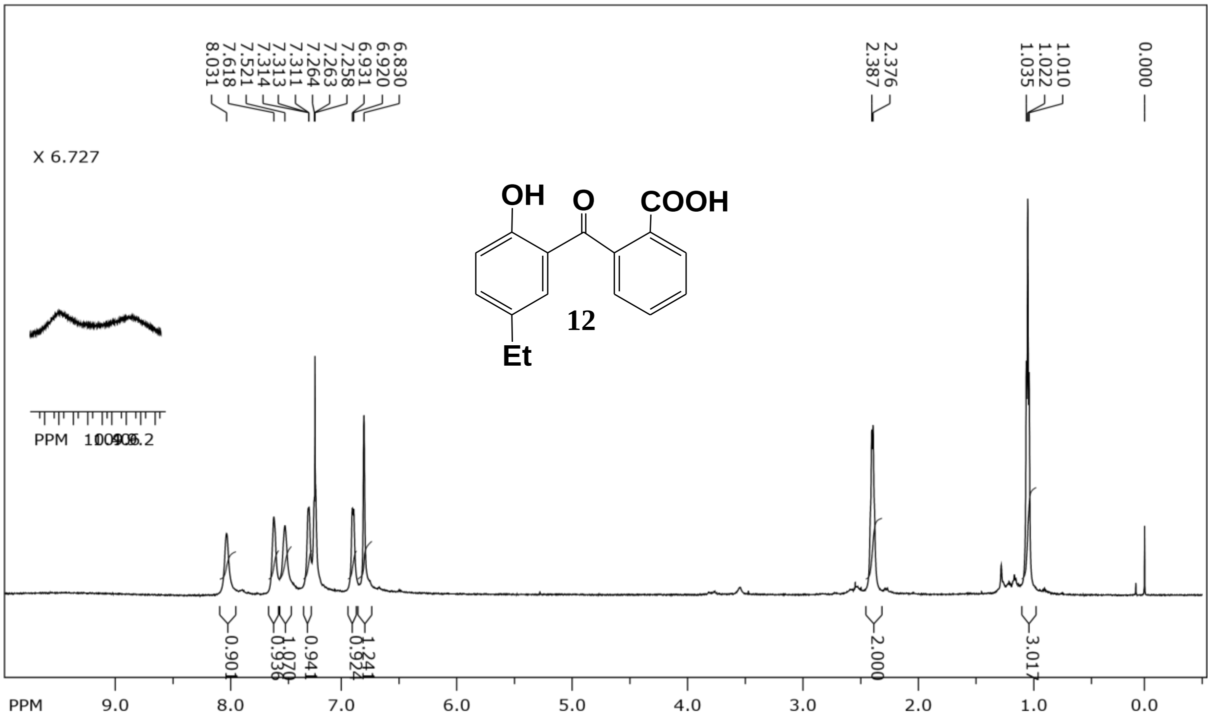

Figure S11. ^{1}H NMR (300 MHz, $CDCl_3$) spectrum of **12**

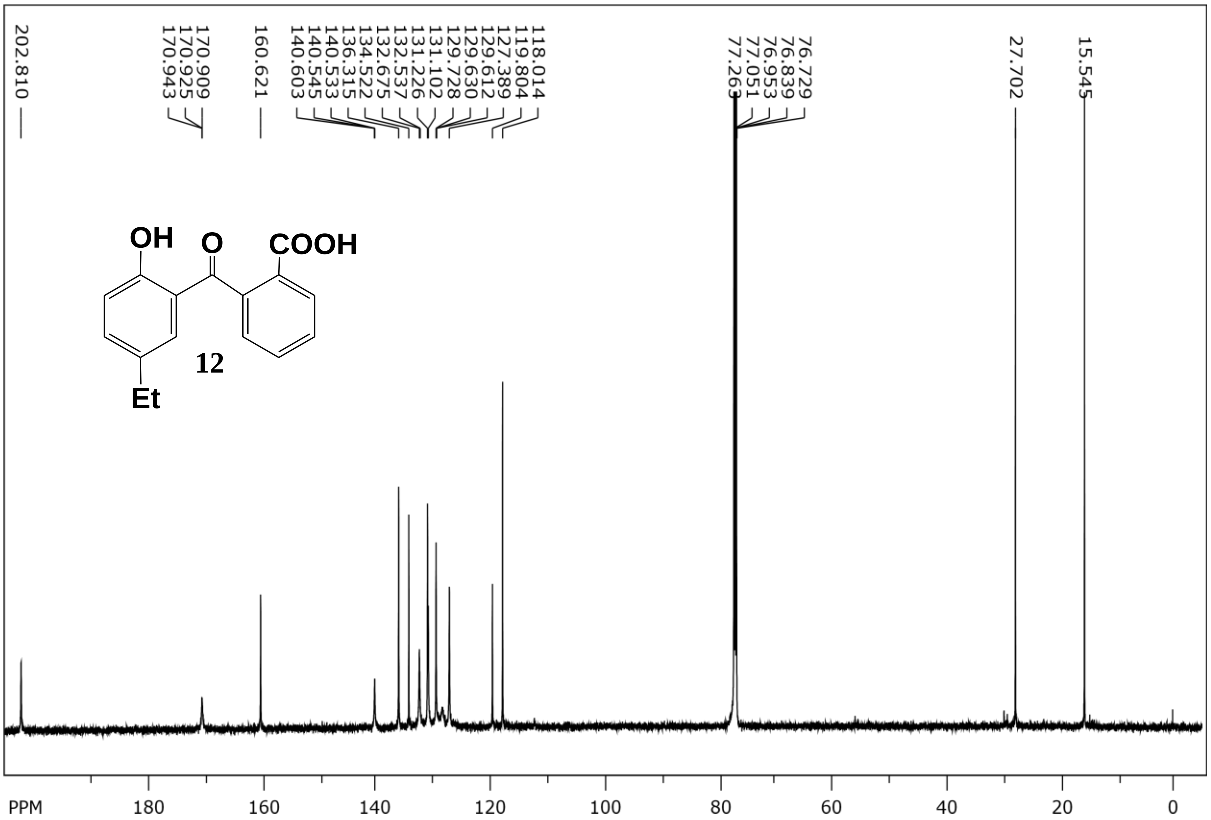

Figure S12. ^{13}C NMR (300 MHz, $CDCl_3$) spectrum of **12**

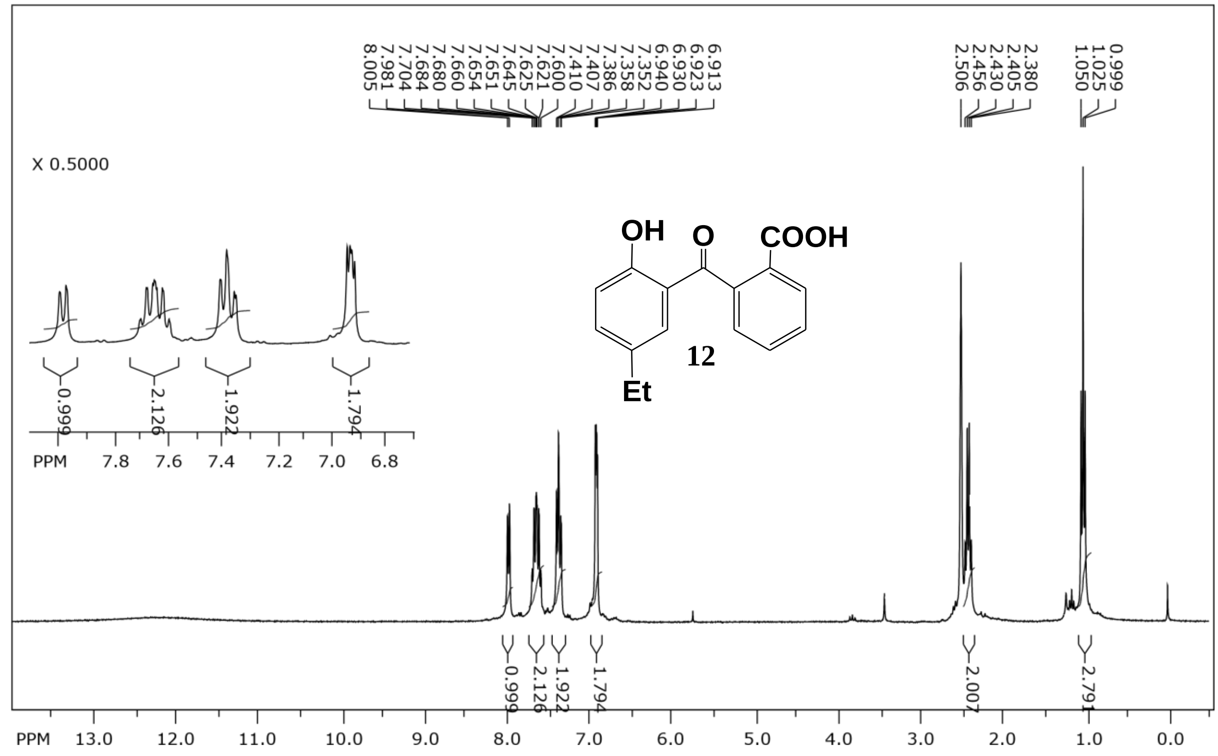

Figure S13. ^{1}H NMR (300 MHz, DMSO-d_6) spectrum of **12**

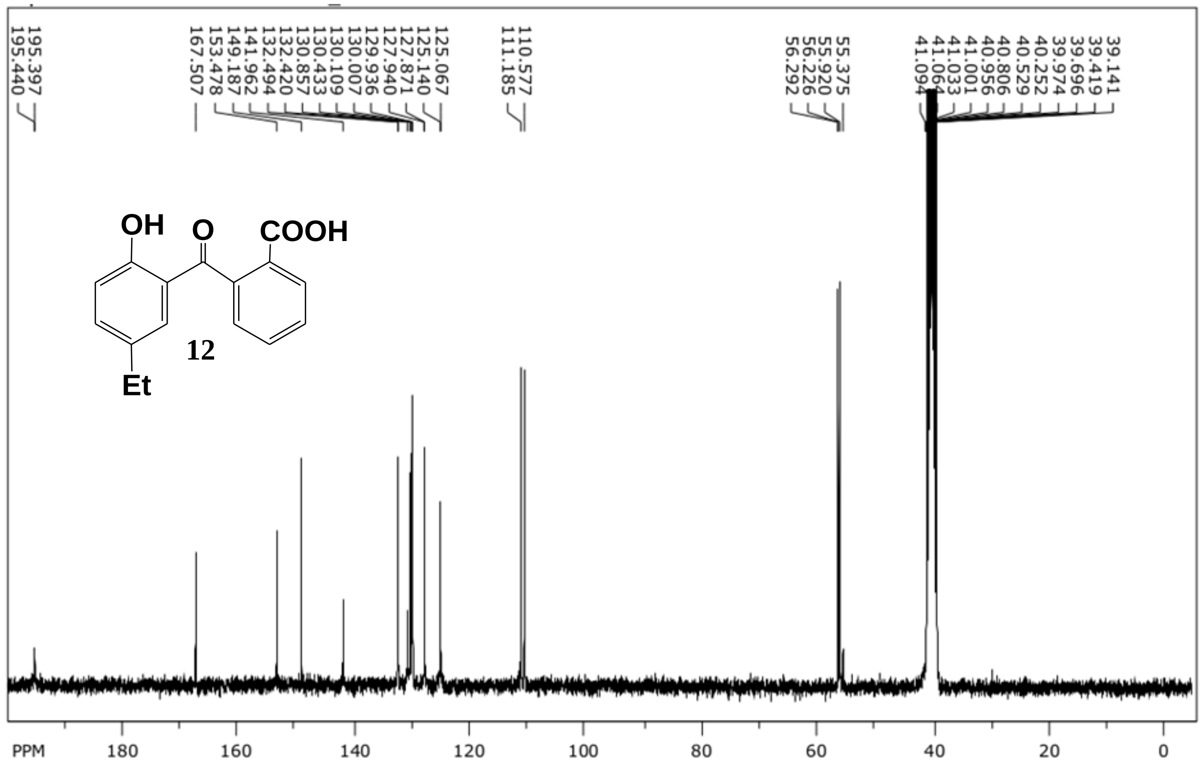

Figure S14. ^{13}C NMR (300 MHz, DMSO-d_6) spectrum of **12**

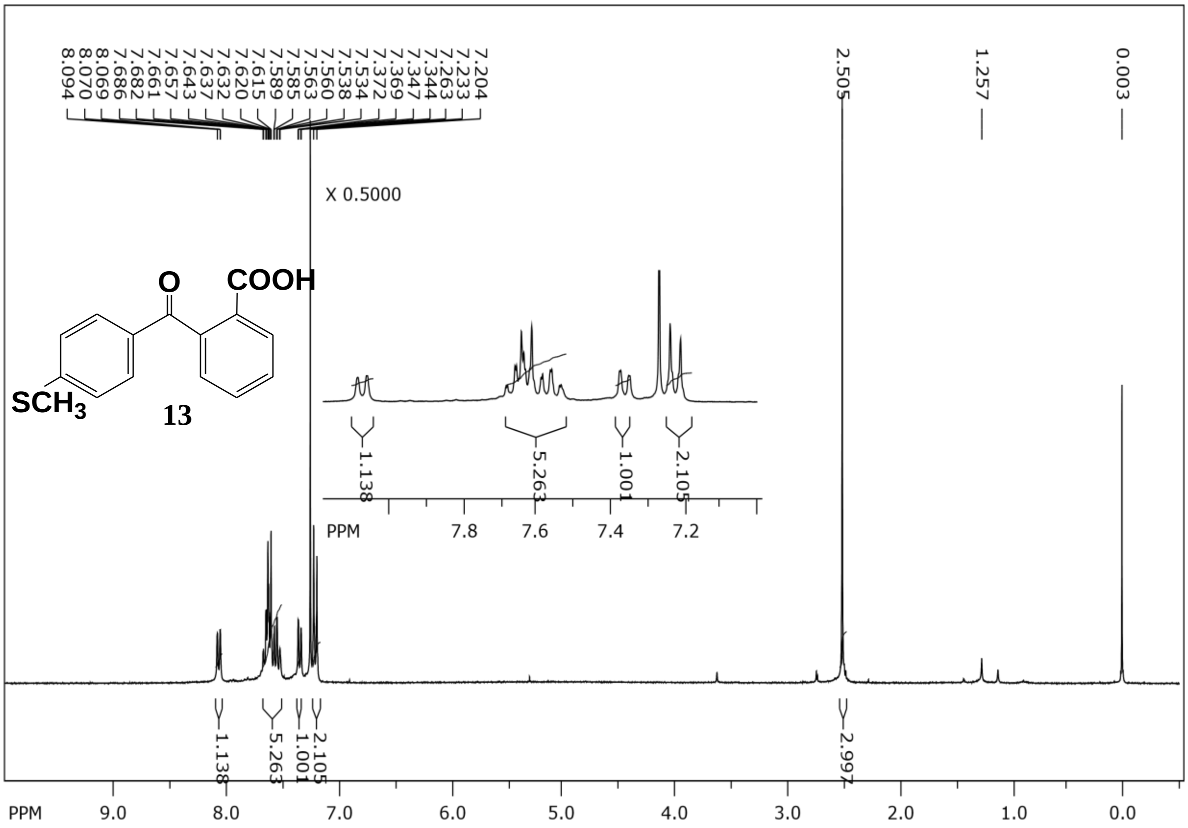

Figure S15. ^{1}H NMR (300 MHz, $CDCl_3$) spectrum of **13**

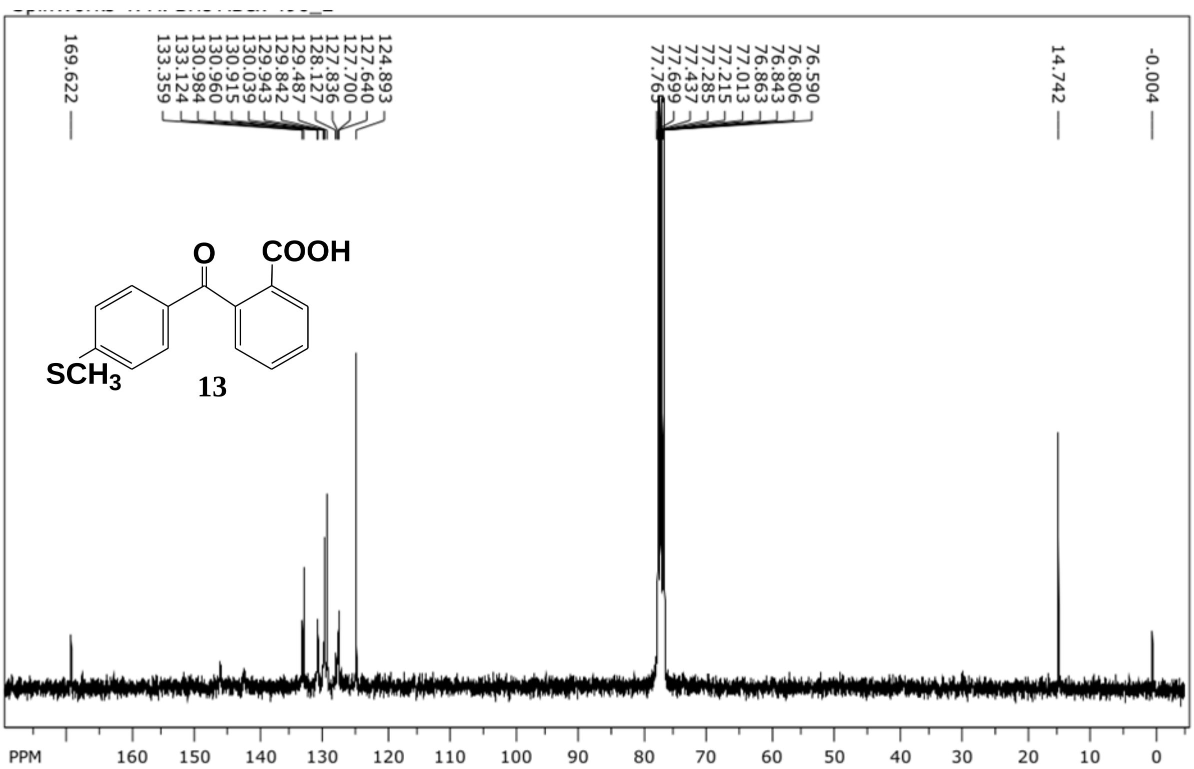

Figure S16. ^{13}C NMR (300 MHz, $CDCl_3$) spectrum of **13**

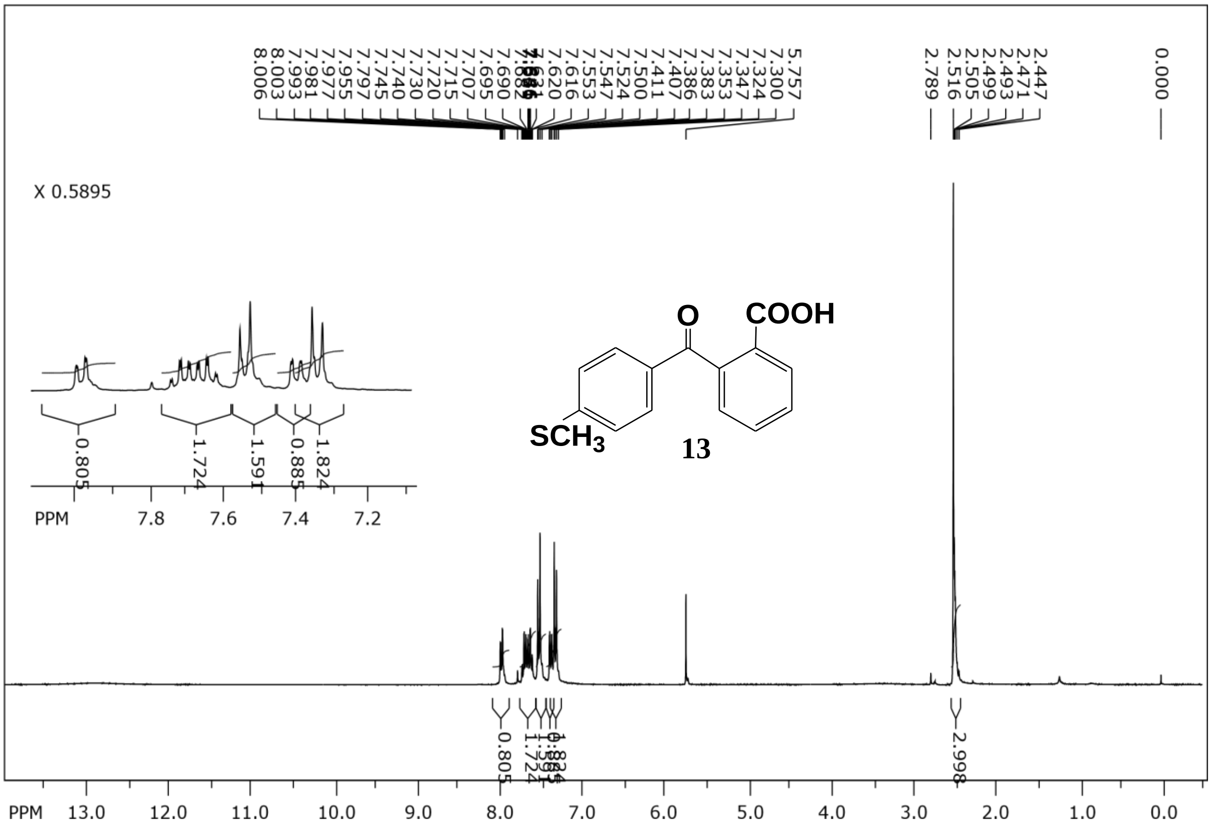

Figure S17. ^{1}H NMR (300 MHz, DMSO-d_6) spectrum of **13**

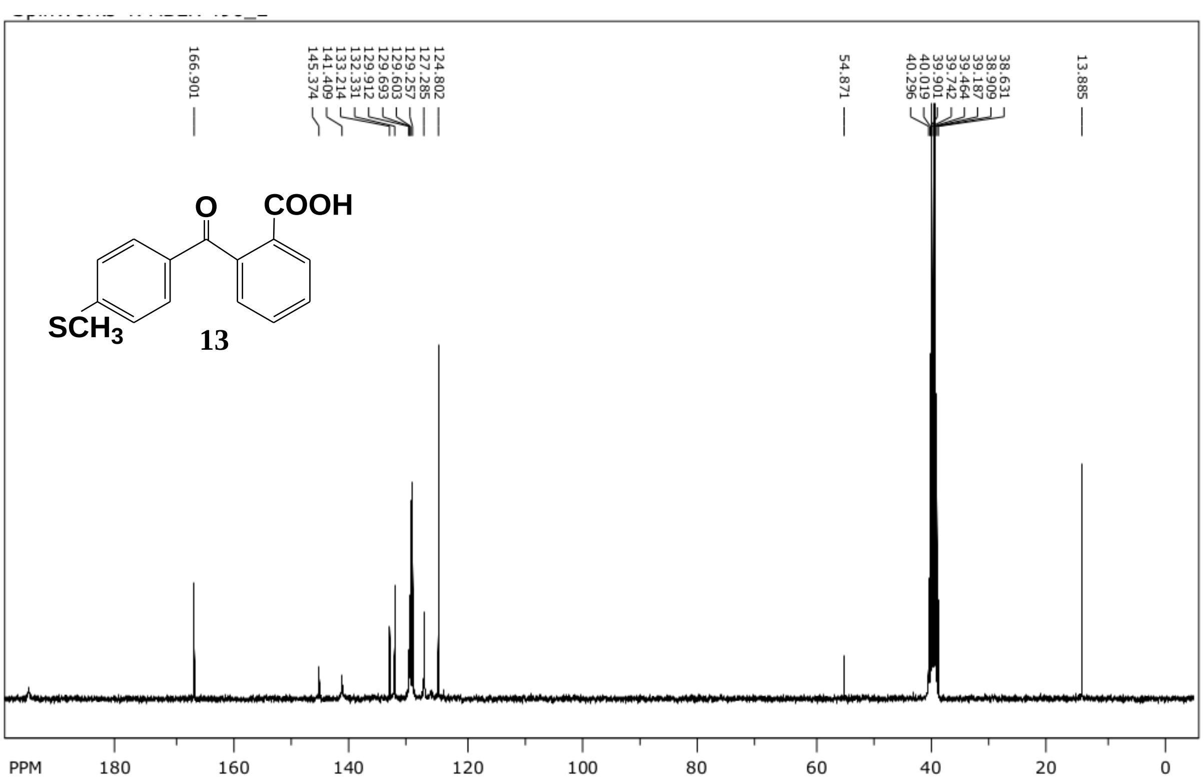

Figure S18. ^{13}C NMR (300 MHz, DMSO-d_6) spectrum of **13**

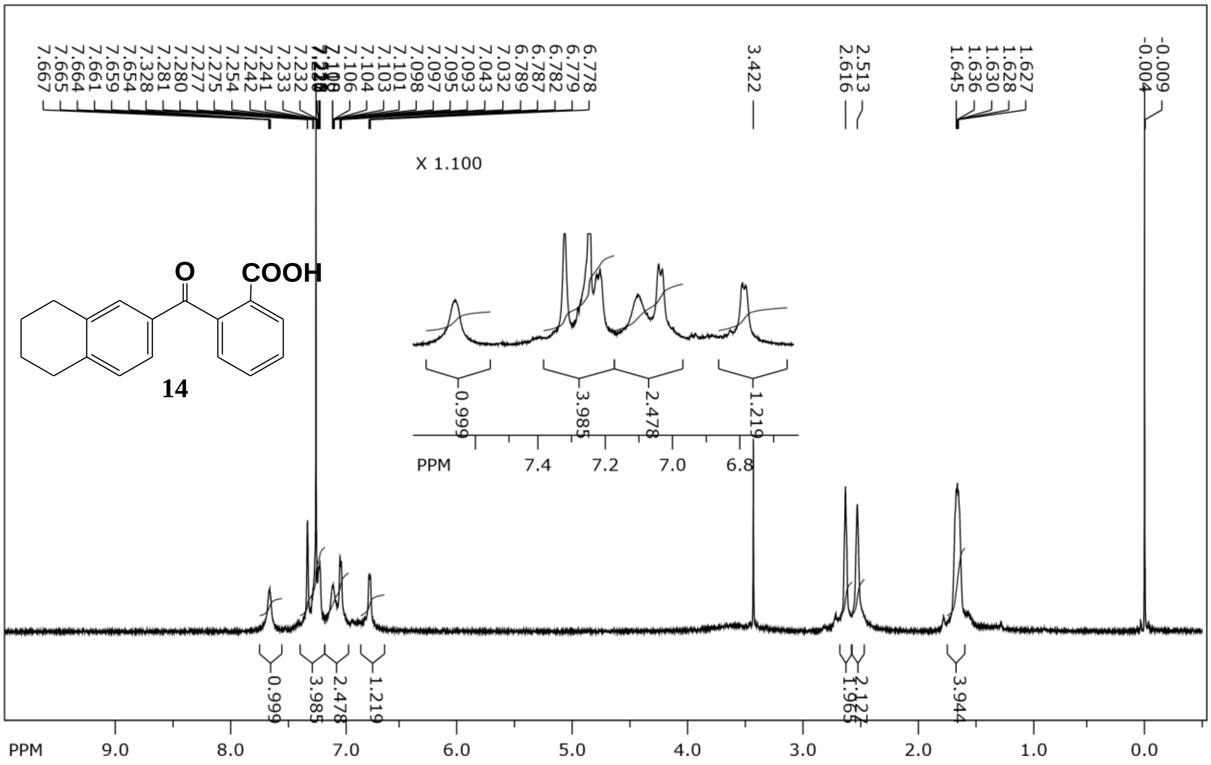

Figure S19. ^{1}H NMR (300 MHz, $CDCl_3$) spectrum of **14**

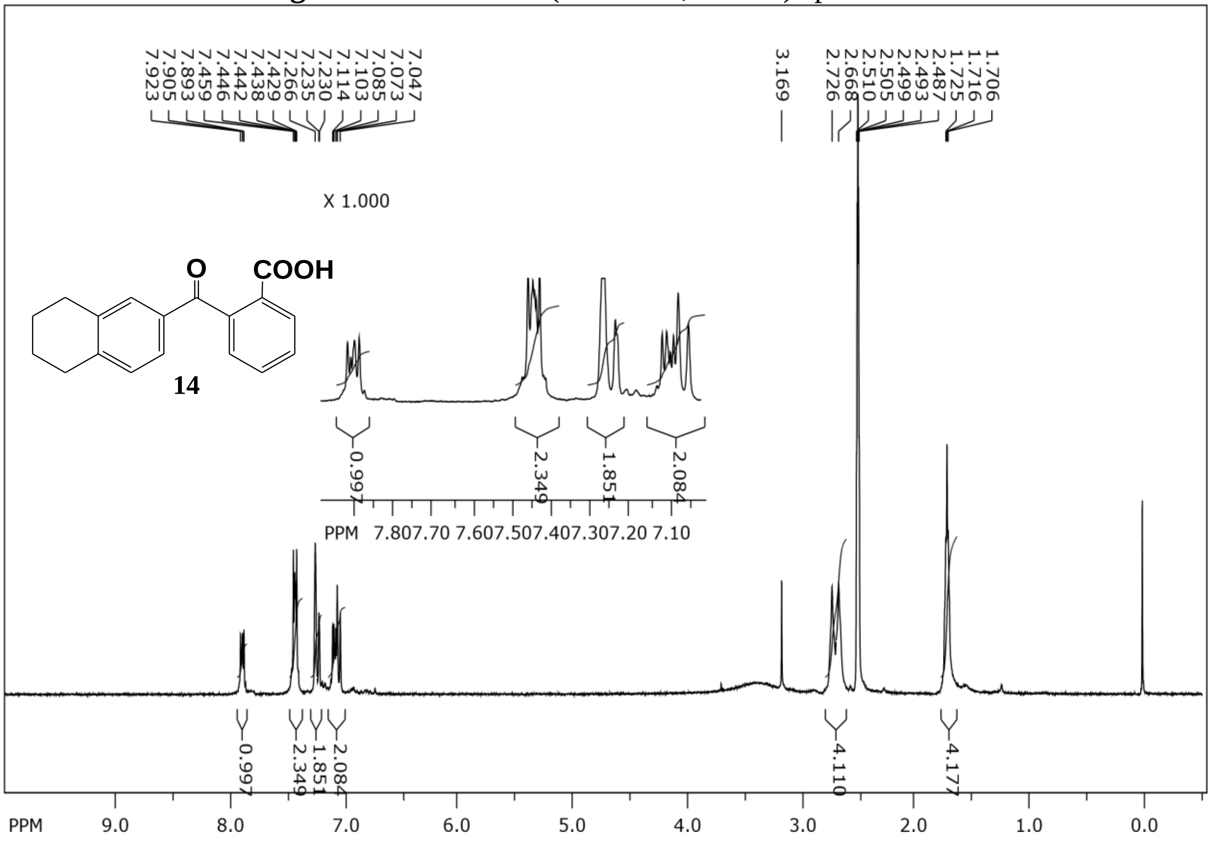

Figure S20. ^{1}H NMR (300 MHz, DMSO-d_6) spectrum of **14**

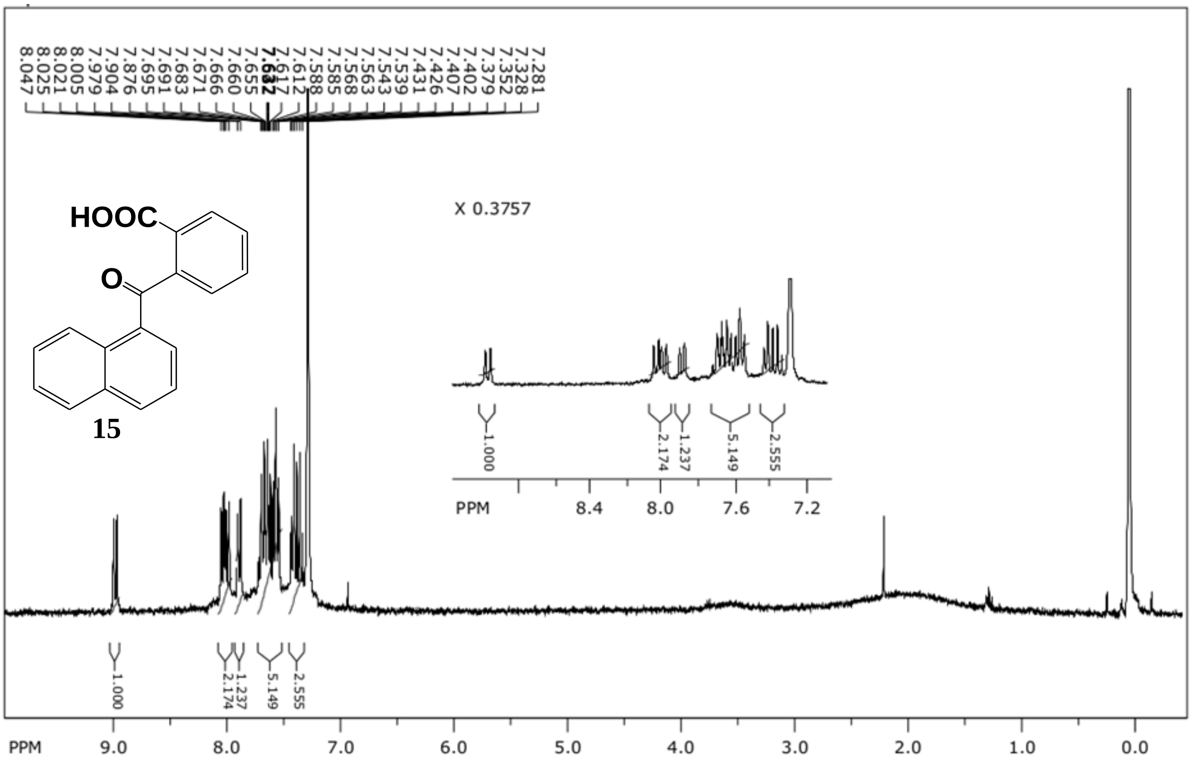

Figure S21. ^{1}H NMR (300 MHz, $CDCl_3$) spectrum of **15**

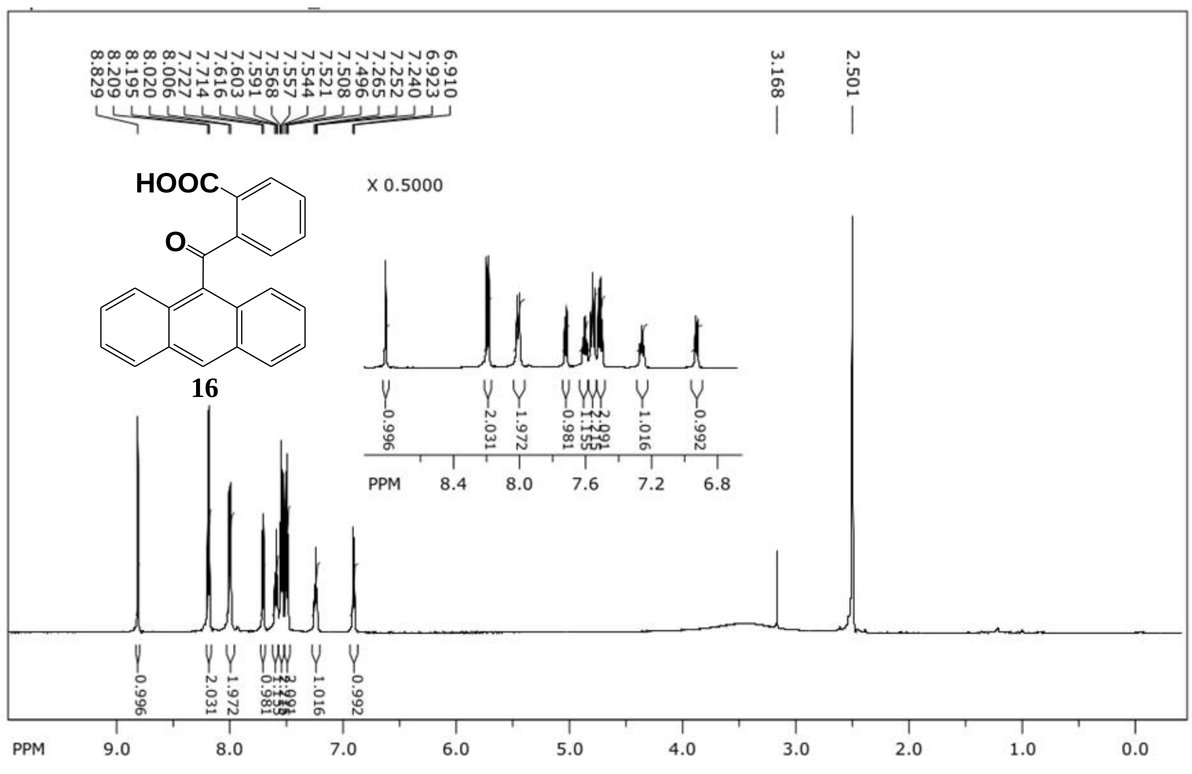

Figure S22. ^{1}H NMR (300 MHz, DMSO-d_6) spectrum of **16**

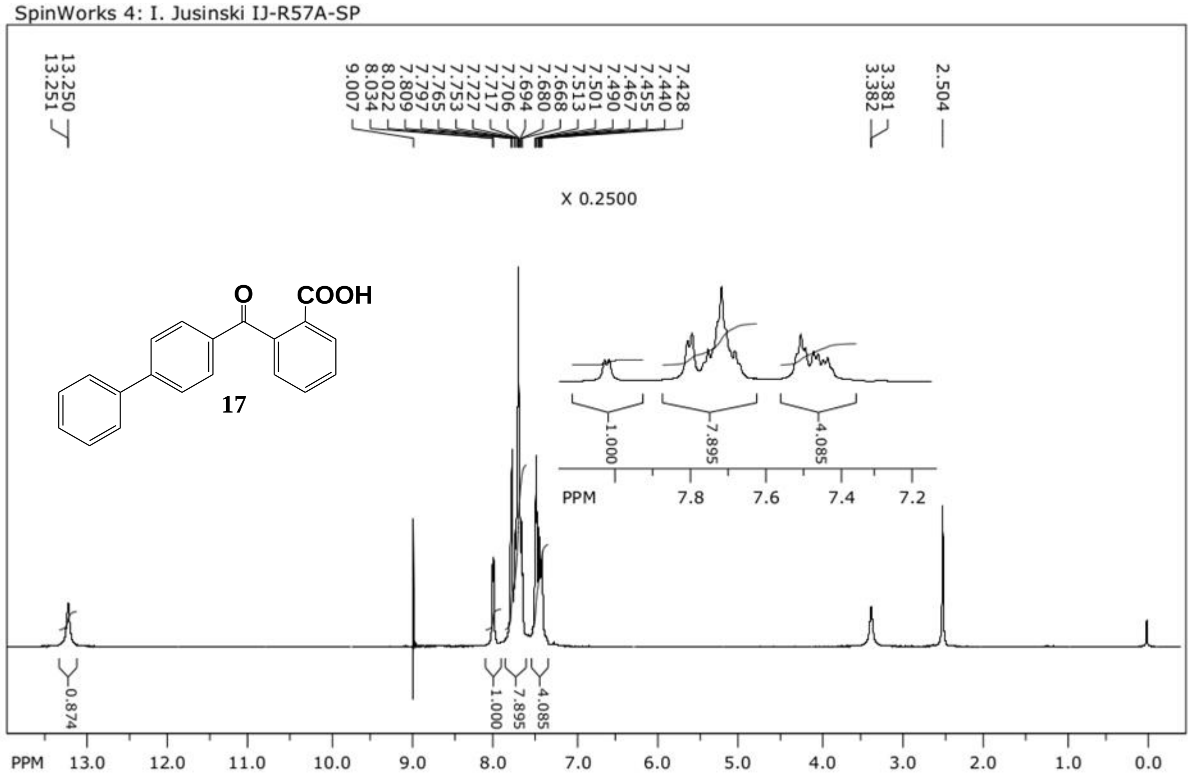

Figure S23. ^{1}H NMR (300 MHz, DMSO-d_6) spectrum of **17**

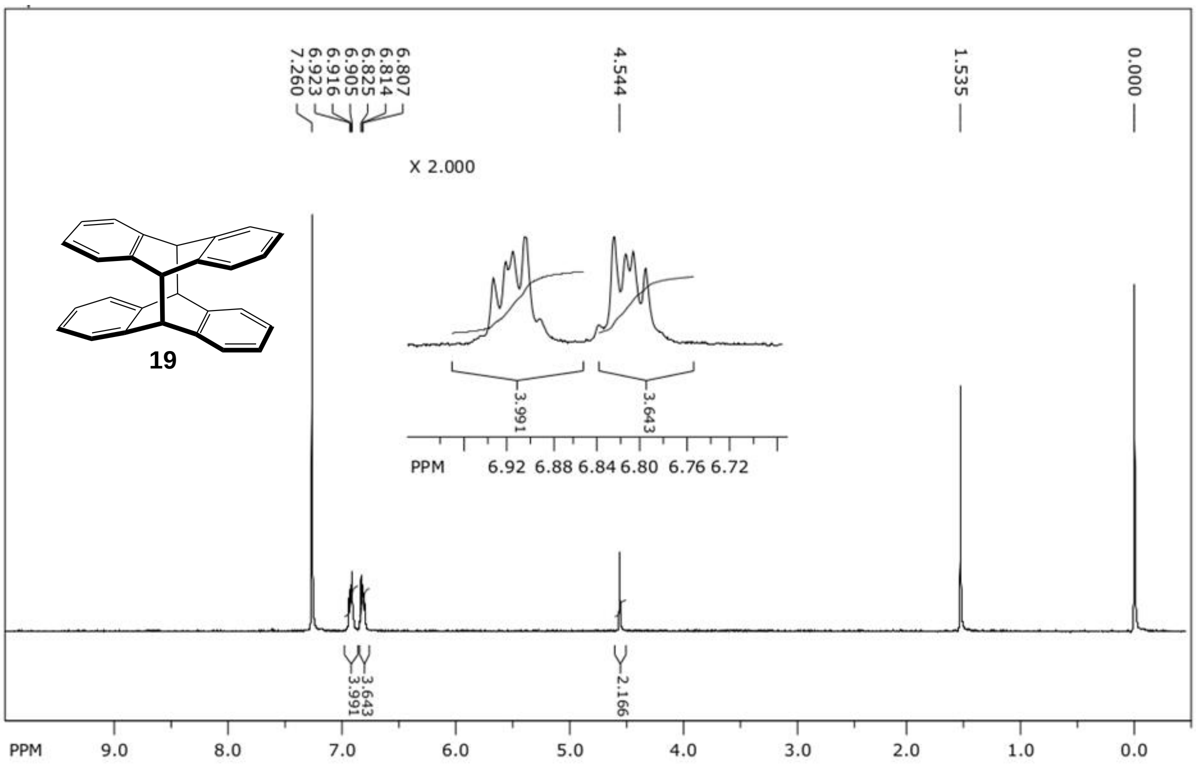

Figure S24. ^{1}H NMR (300 MHz, $CDCl_3$) spectrum of **19**

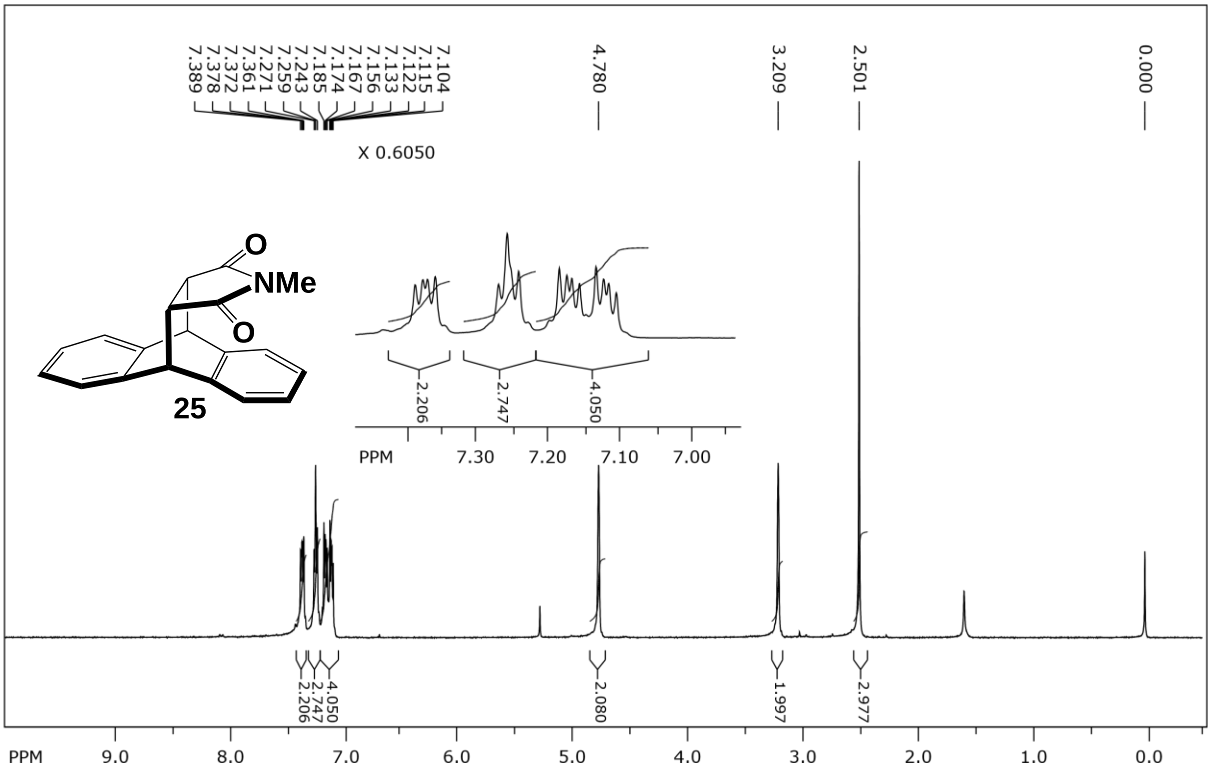

Figure S25. ^{1}H NMR (300 MHz, $CDCl_3$) spectrum of **25**

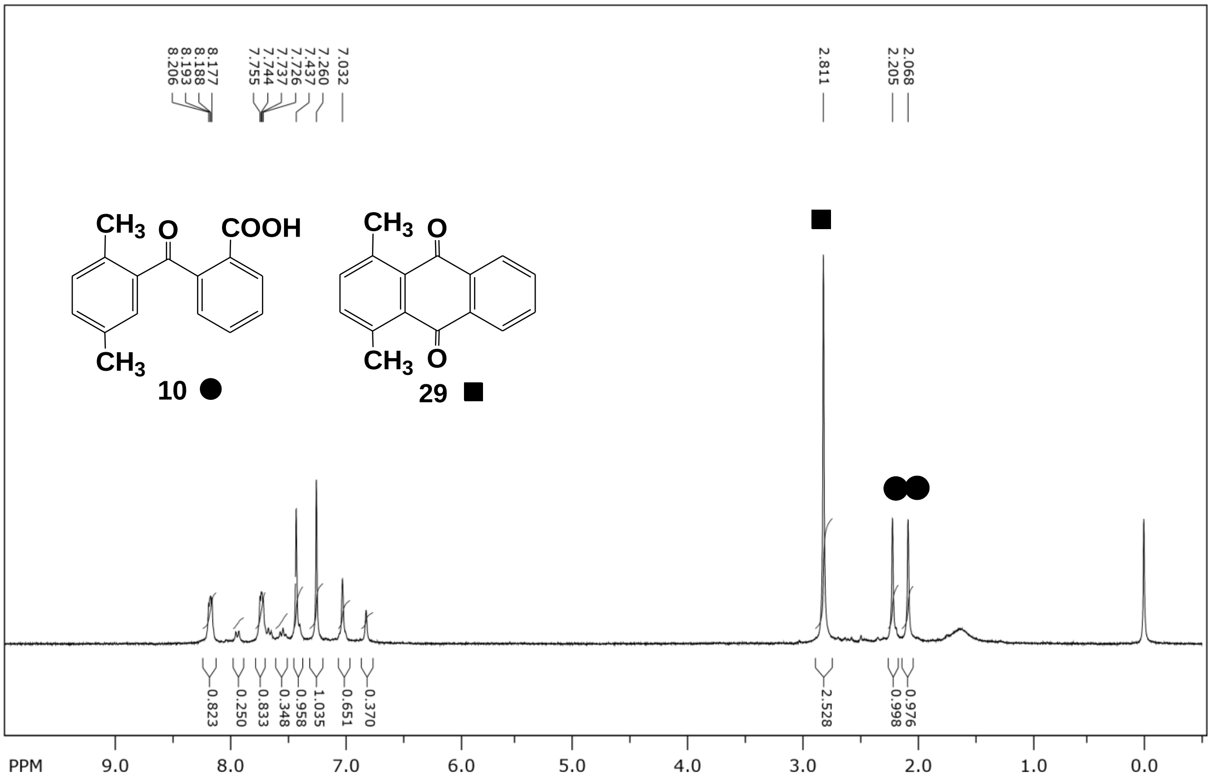

Figure S26. ^{1}H NMR (300 MHz, $CDCl_3$) spectrum of mixture of **10** and **29**

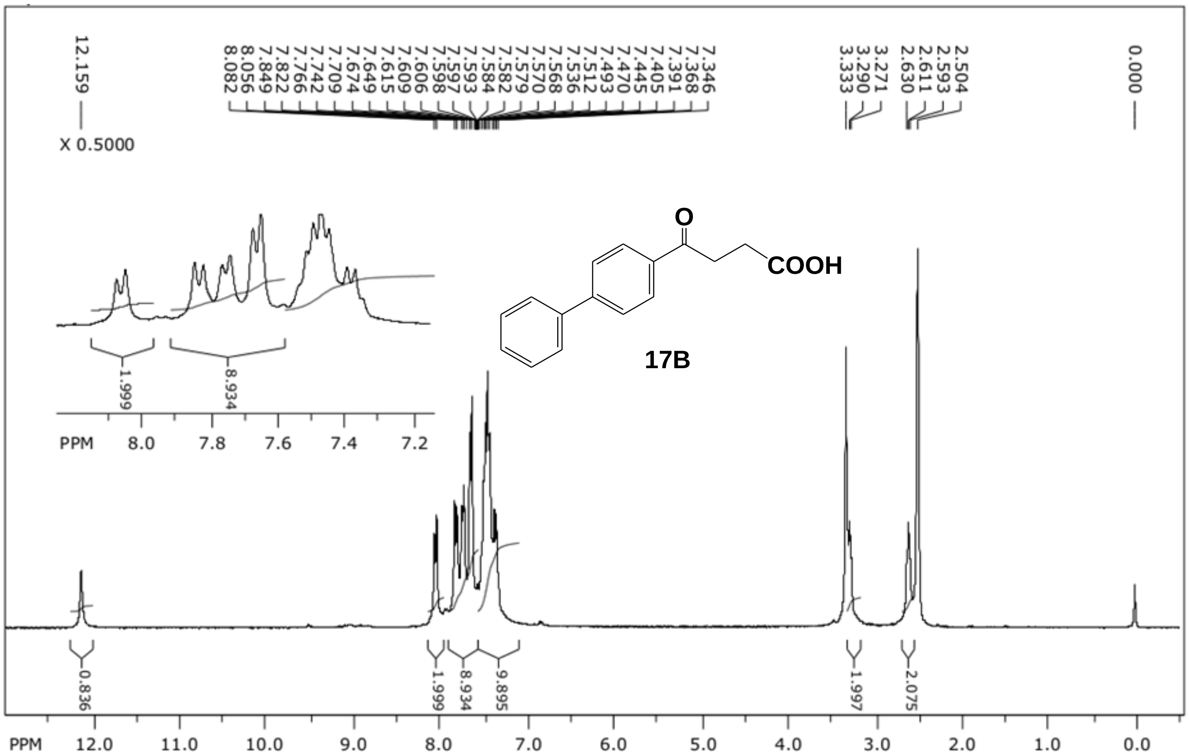

Figure S27. ^{1}H NMR (300 MHz, DMSO-d_6) spectrum of **17B**

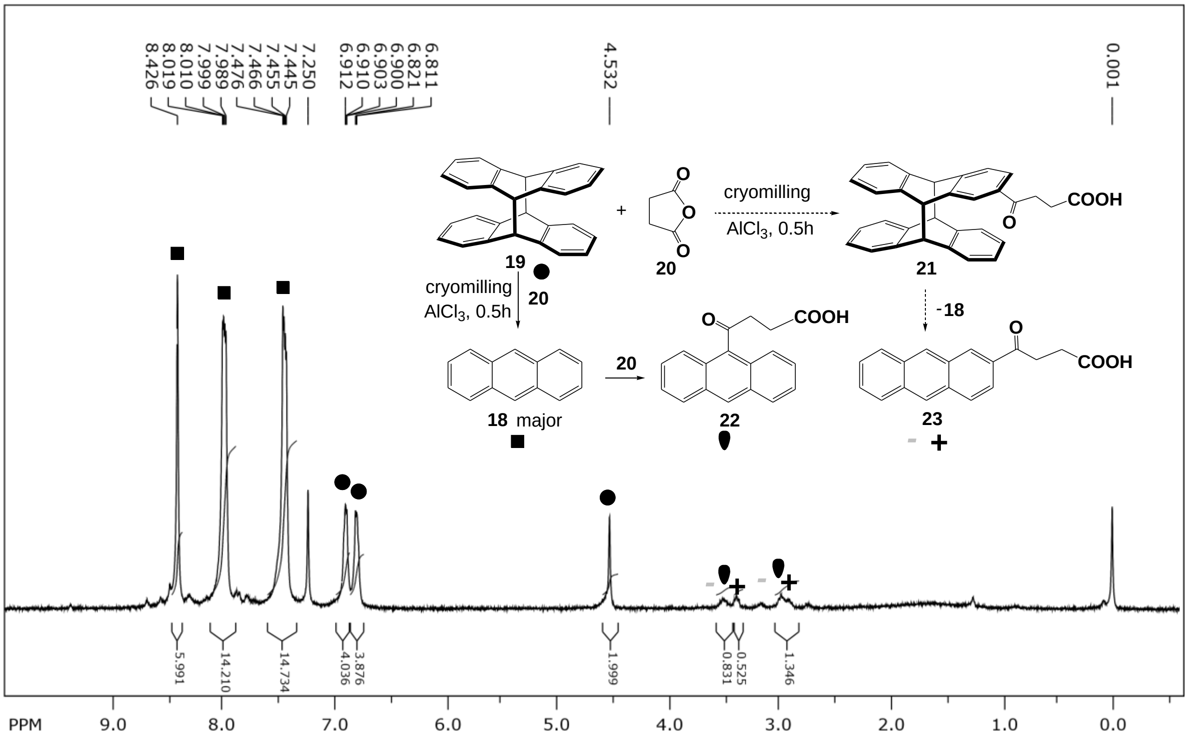

Figure S28. ^{1}H NMR (300 MHz, $CDCl_3$) spectrum of cryomilling mixture of **18-23**

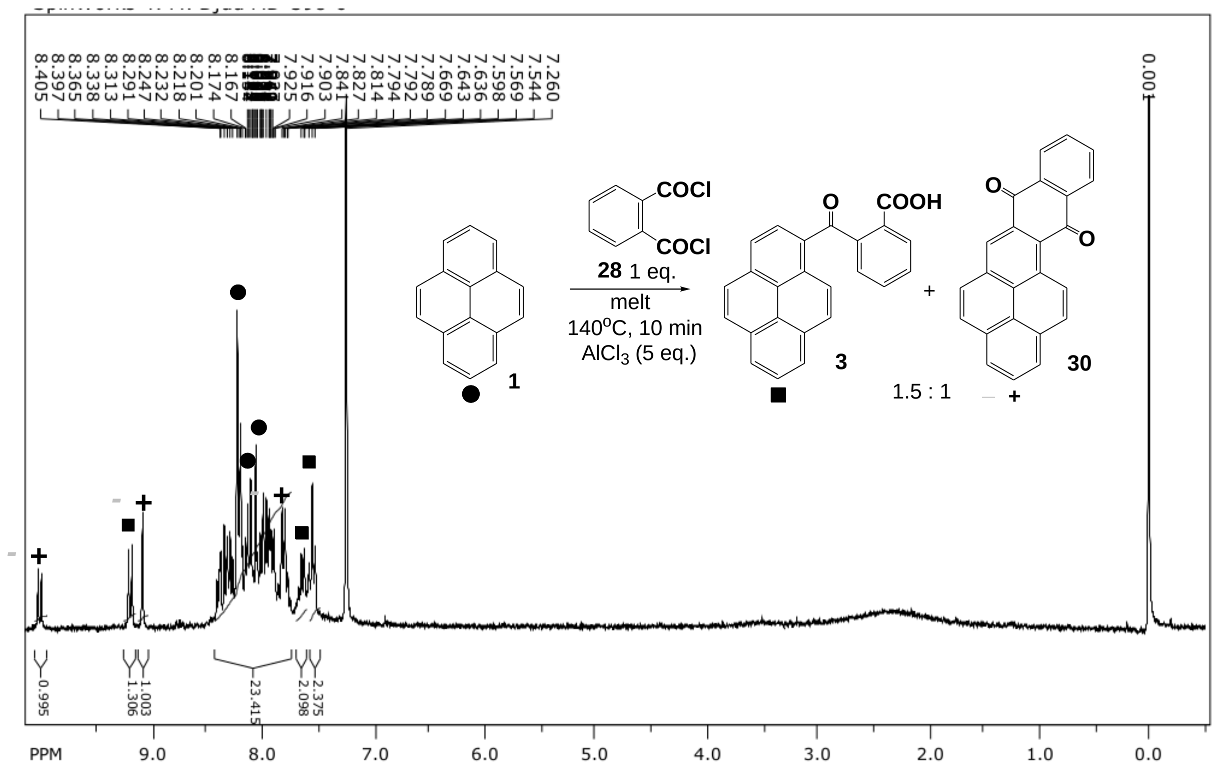

Figure S29. ^{1}H NMR (300 MHz, $CDCl_3$) spectrum of melt reaction of pyrene **1** with **28**

IR spectra

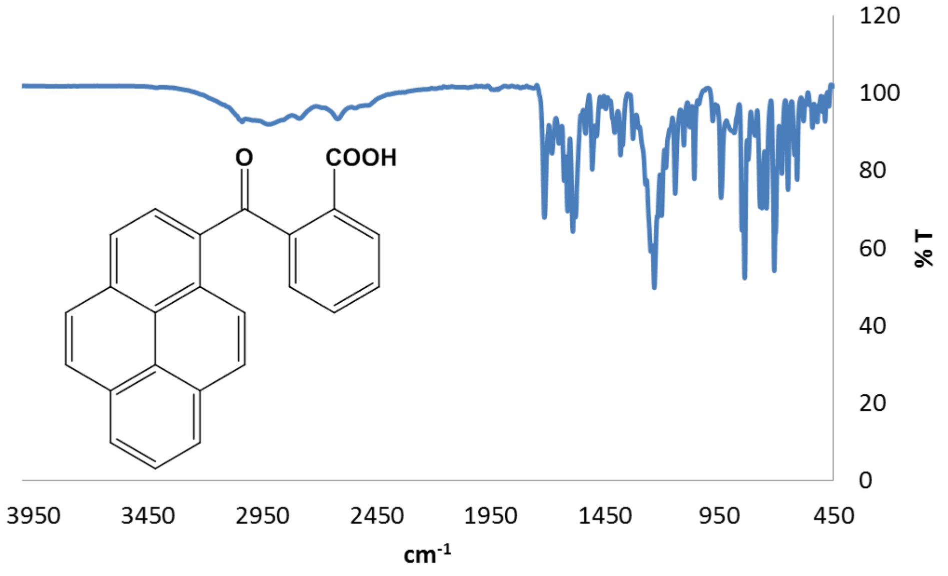

Figure S30. IR-ATR spectrum of **3**

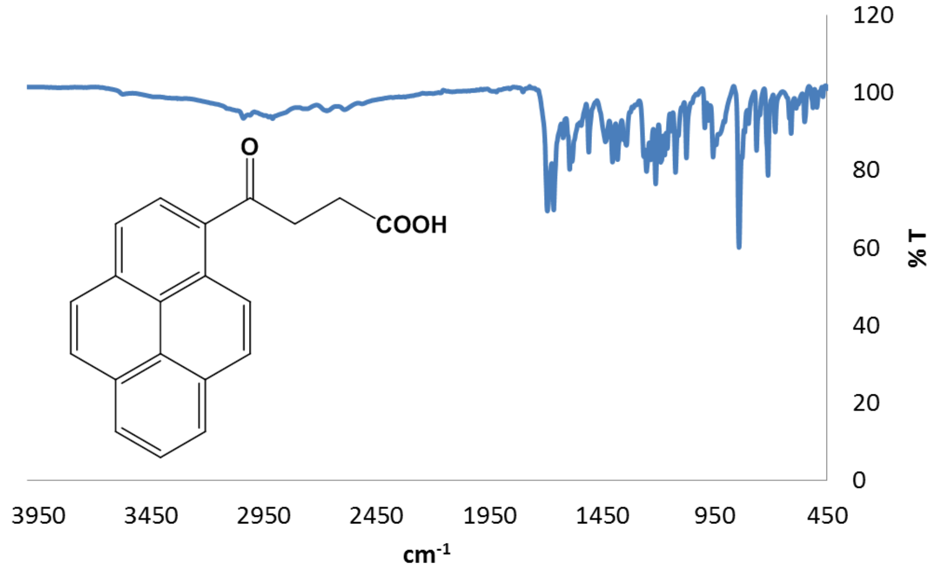

Figure S31. IR-ATR spectrum of **4**

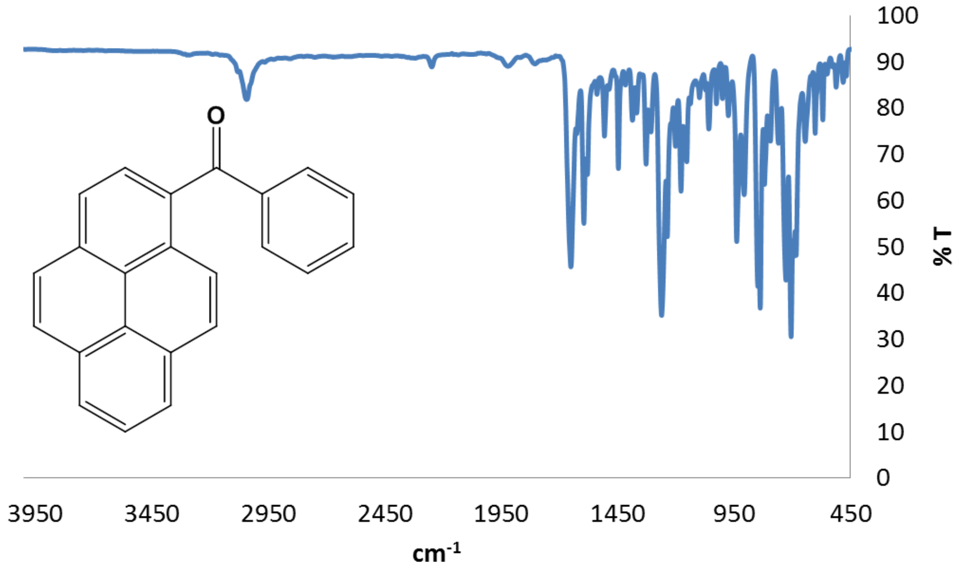

Figure S32. IR-ATR spectrum of **5**

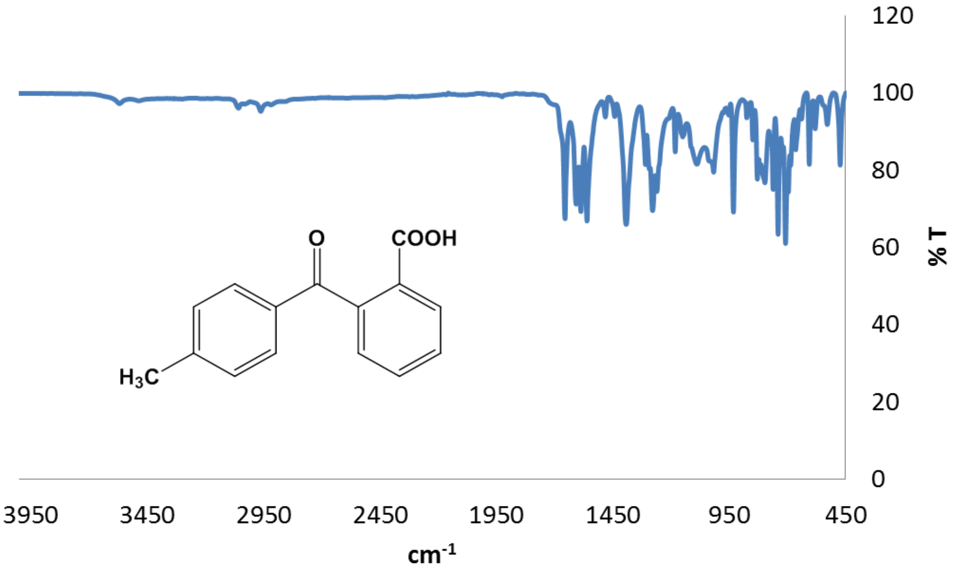

Figure S33. IR-ATR spectrum of **8**

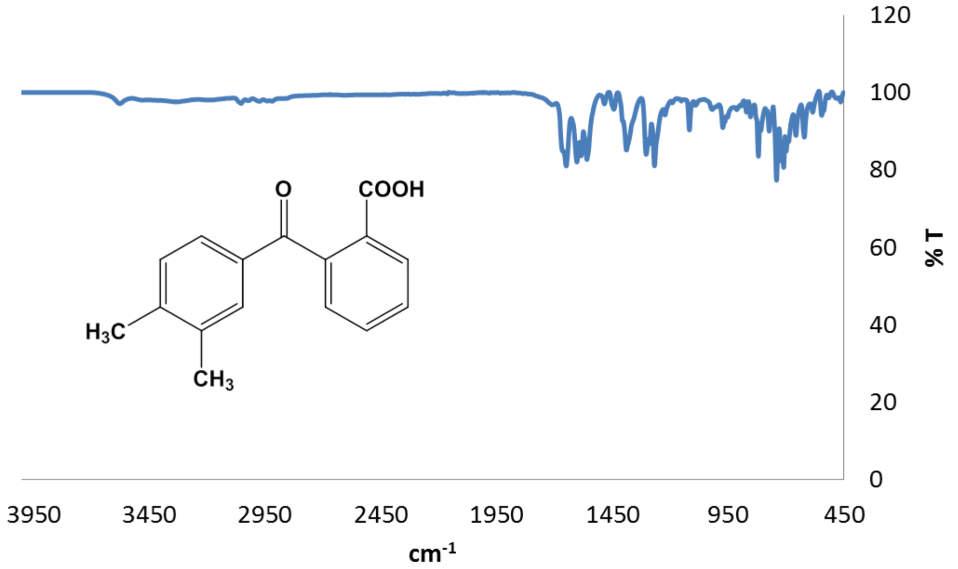

Figure S34. IR-ATR spectrum of **9**

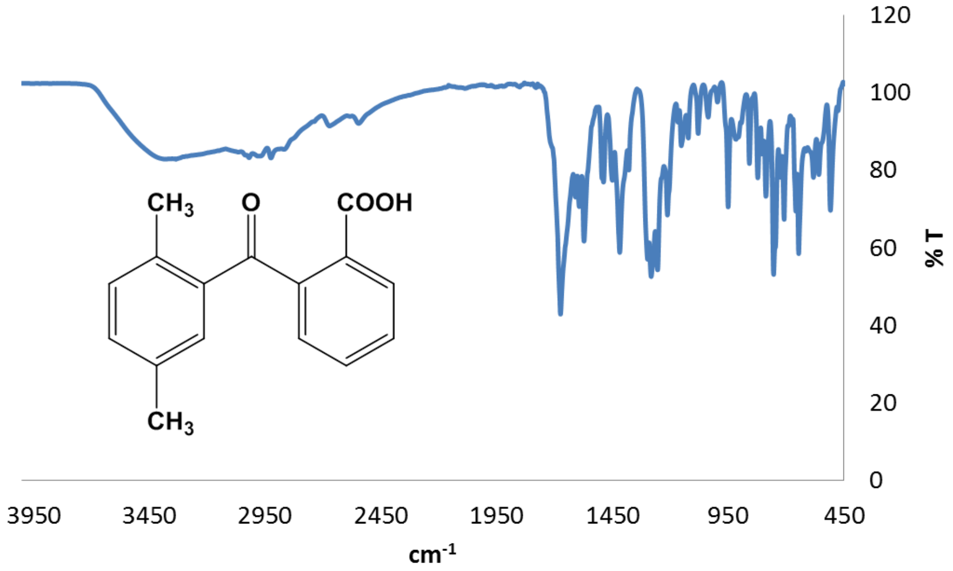

Figure S35. IR-ATR spectrum of **10**

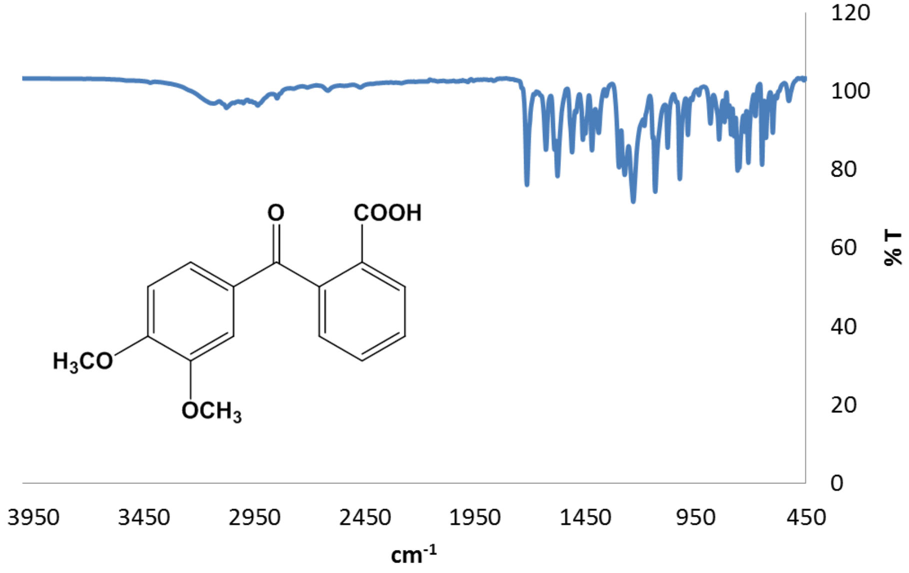

Figure S36. IR-ATR spectrum of **11**

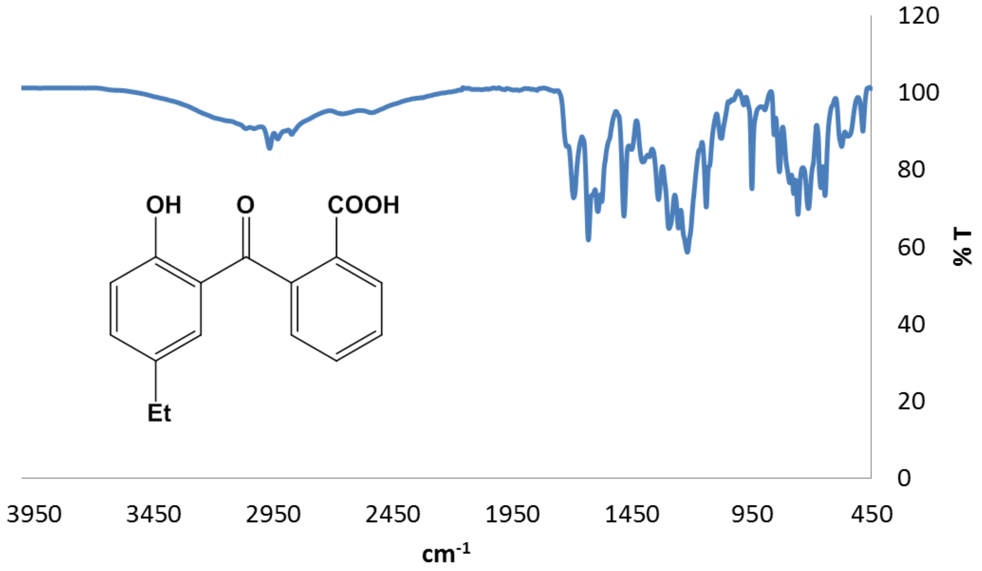

Figure S37. IR-ATR spectrum of **12**

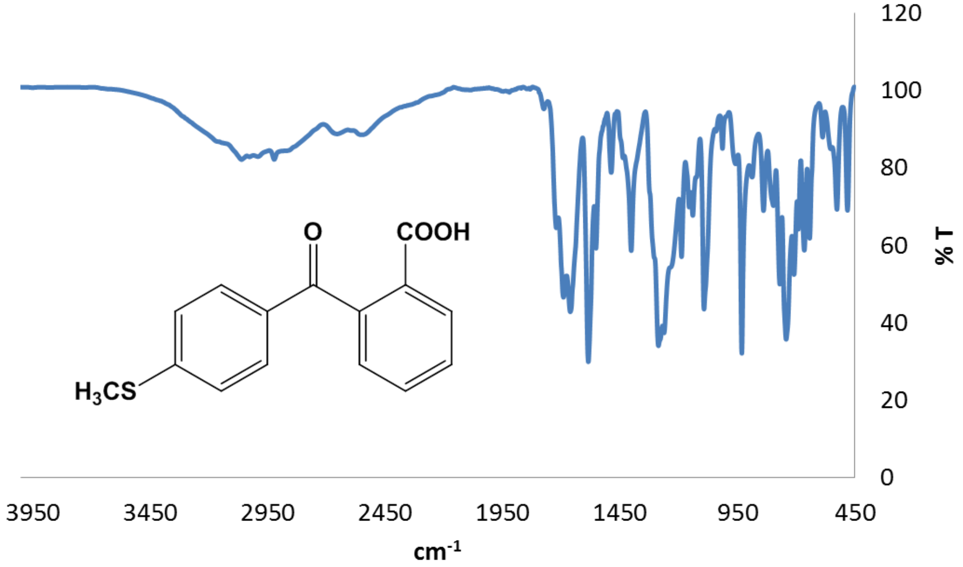

Figure S38. IR-ATR spectrum of **13**

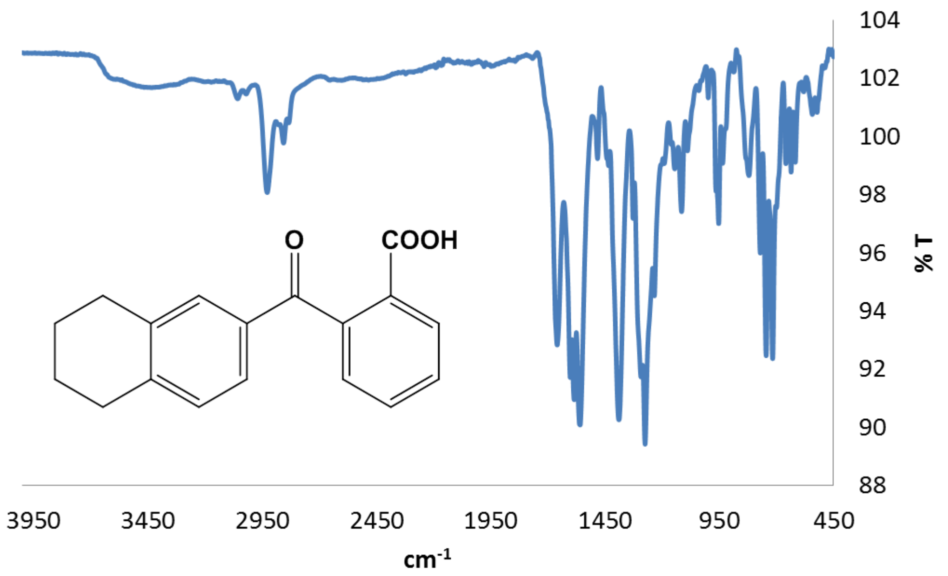

Figure S39. IR-ATR spectrum of **14**

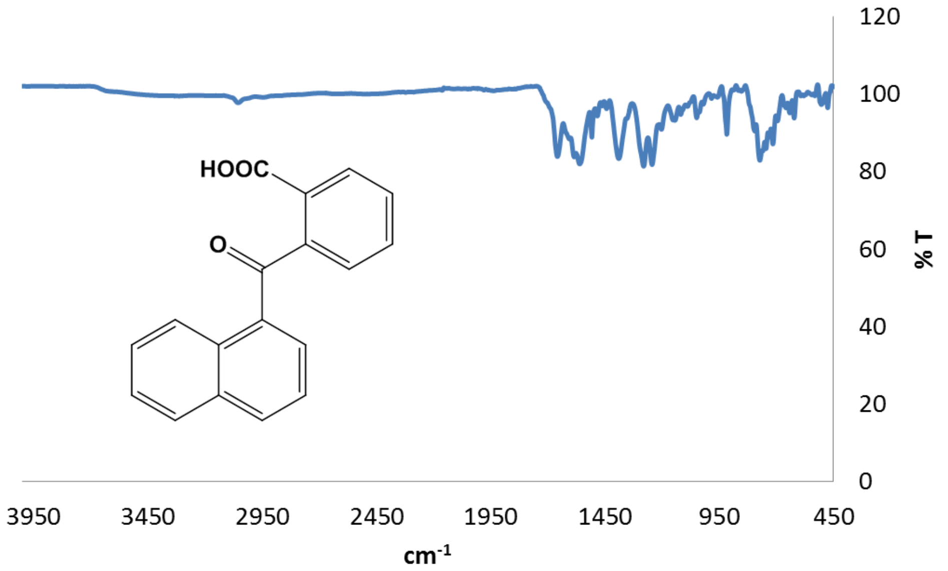

Figure S40. IR-ATR spectrum of **15**

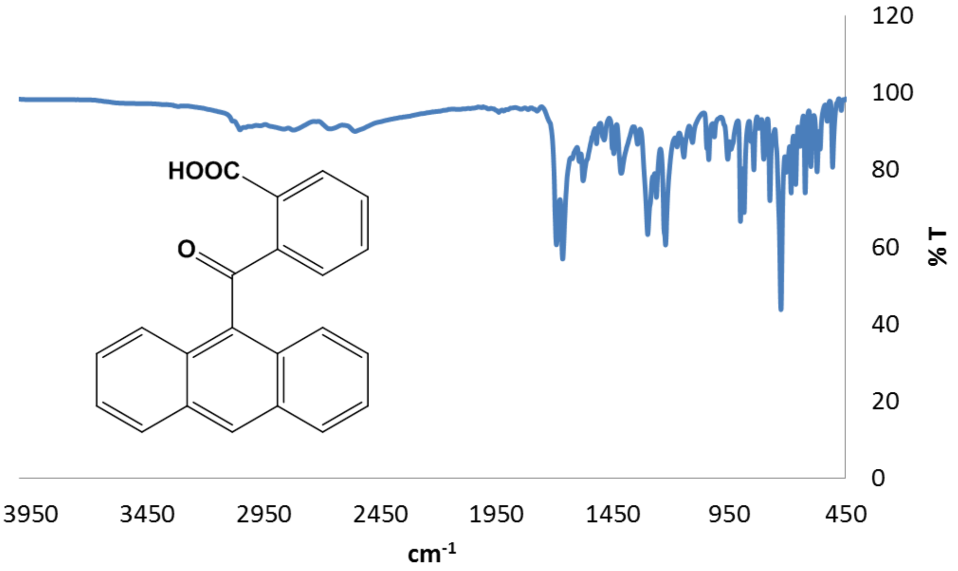

Figure S41. IR-ATR spectrum of **16**

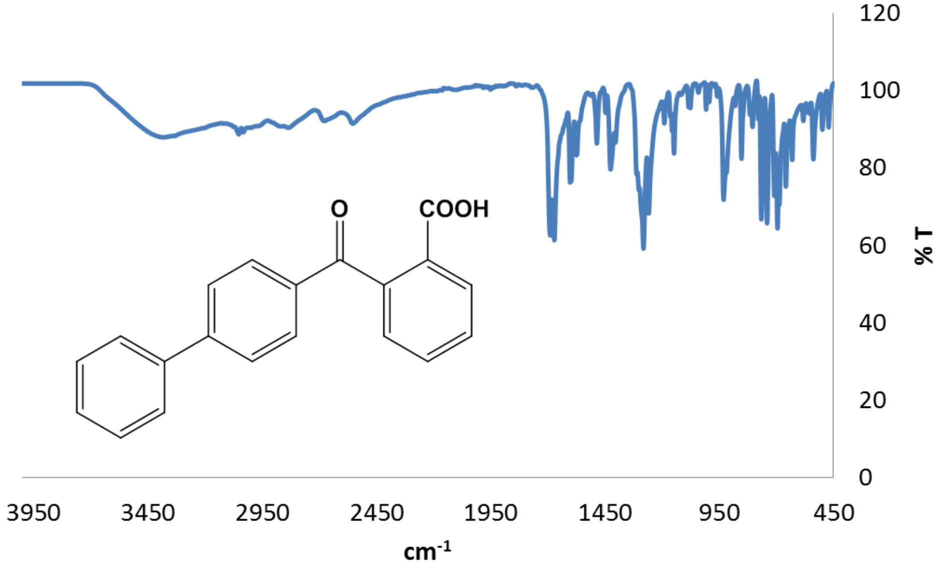

Figure S42. IR-ATR spectrum of **17**

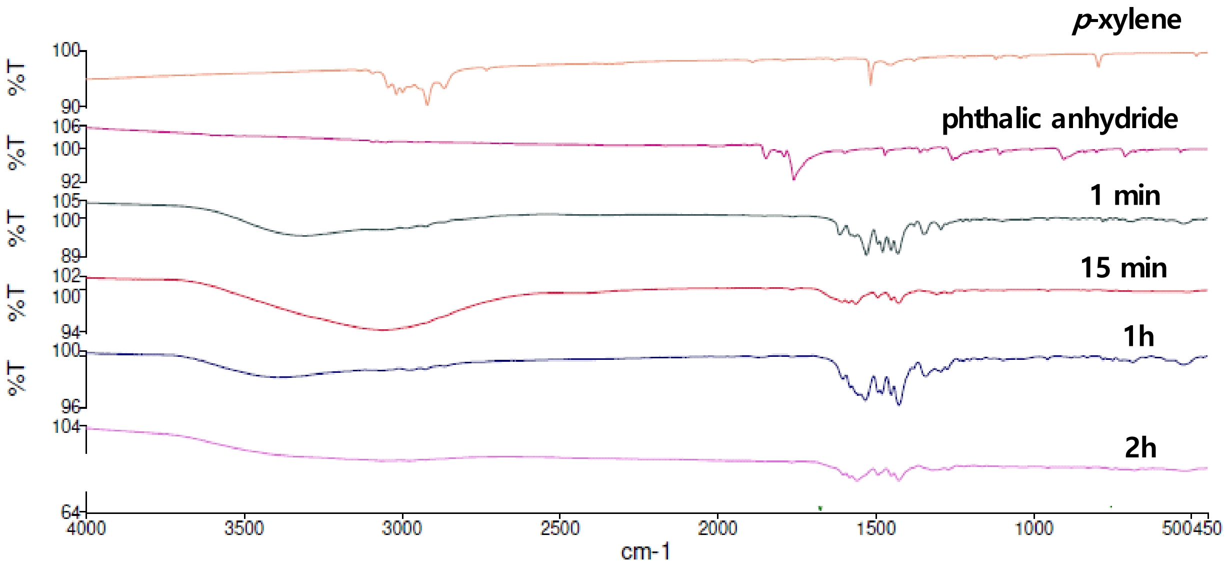

Figure S43. Ex situ IR spectroscopy of *p*-xylene FC reaction with phthalic anhydride

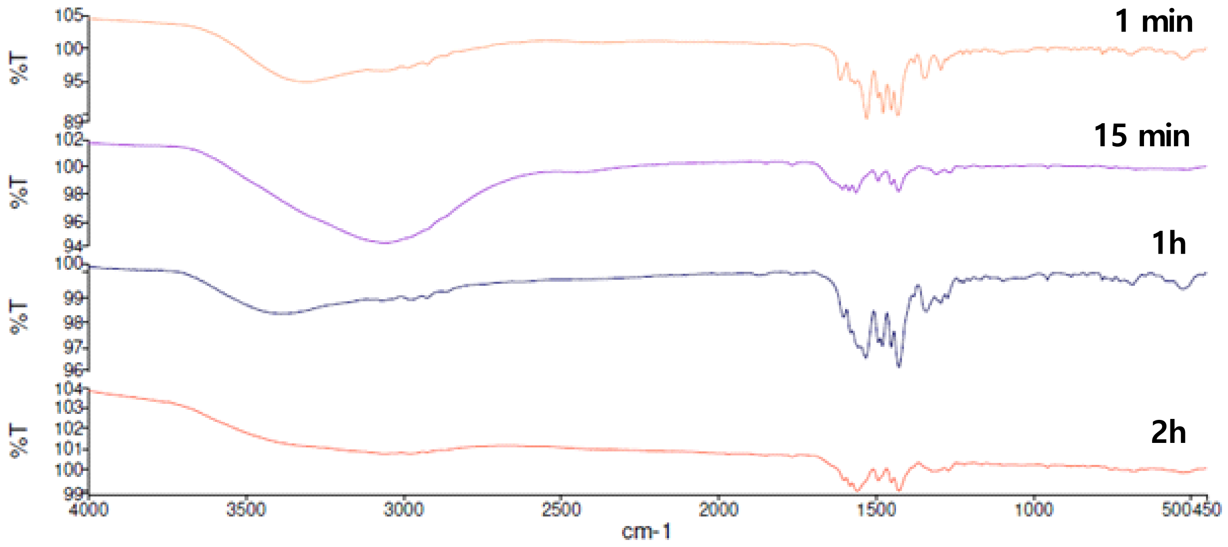

Figure S44. Ex situ IR spectroscopy of *p*-xylene FC reaction with phthalic anhydride (enlarged)

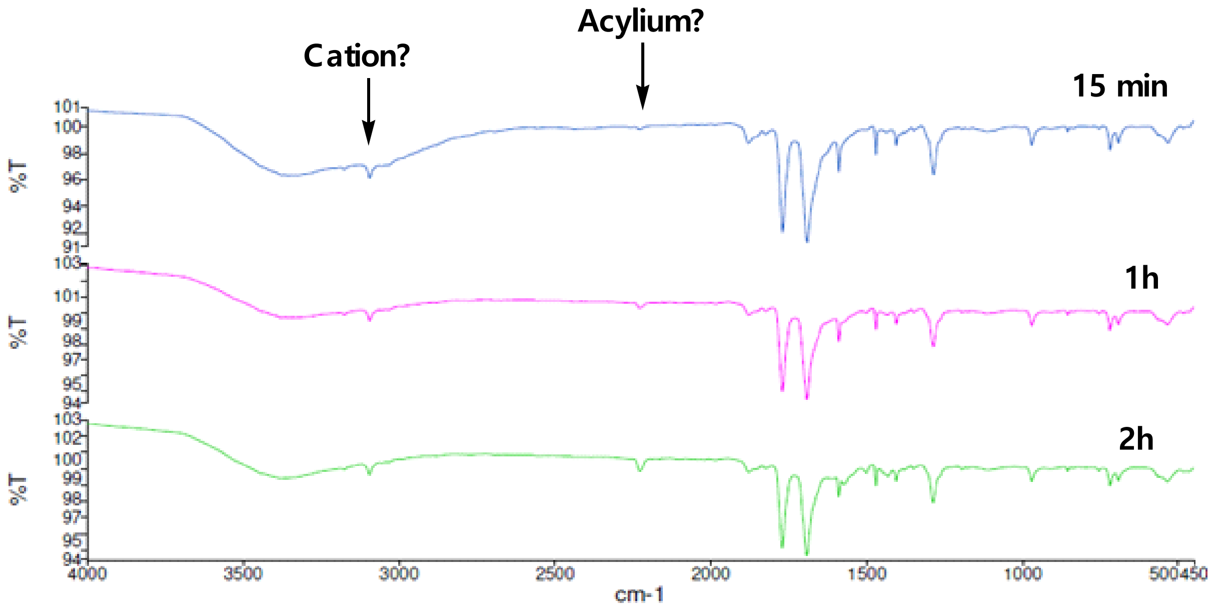

Figure S45. Ex situ IR spectra of phthalic anhydride complexation with $AlCl_3$

Computational details

All computational studies reported in this work were performed on dual core Opteron 240 personal computer under Linux operating system. Geometries of all species were optimized using Becke, three-parameter, Lee-Yang-Parr[24] exchange-correlation functional B3LYP/6-31G* method as implemented in Gaussian 03 suite of programs.[25] All minima were verified by vibrational analysis (no imaginary frequencies were obtained). Zero point vibrational energies (ZPVE) were corrected using scaling factor *f* = 0.9614 according to literature recommendation.[26]

Cartesian coordinates of optimized structures

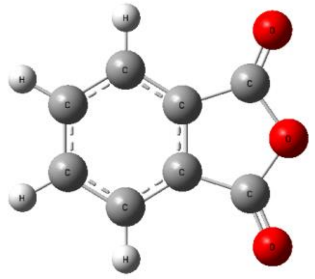

```
Phthalic anhydride B3LYP/6-31G*

0 1
 C                  -2.54928800   -0.70183900    0.00004100
 C                  -1.35304200   -1.42658500   -0.00004300
 C                  -0.17086900   -0.69597500   -0.00005500
 C                  -0.17081300    0.69594800   -0.00007000
```

C	-1.35296900	1.42661600	-0.00006300
C	-2.54925200	0.70188200	0.00004600
H	-3.49713000	-1.23219900	0.00011400
H	-1.34129000	-2.51182300	-0.00001600
H	-1.34111300	2.51184800	-0.00004900
H	-3.49709500	1.23225000	0.00012700
C	1.24171000	-1.14865800	0.00005100
O	1.70697600	-2.25333100	-0.00002900
C	1.24182200	1.14855200	0.00004100
O	1.70692400	2.25333700	-0.00000900
O	2.04270500	0.00002800	0.00005600

```
Zero-point correction=                           0.103549
(Hartree/Particle)
 Thermal correction to Energy=                    0.111188
 Thermal correction to Enthalpy=                  0.112132
 Thermal correction to Gibbs Free Energy=         0.070781
 Sum of electronic and zero-point Energies=           -532.848504
 Sum of electronic and thermal Energies=              -532.840865
 Sum of electronic and thermal Enthalpies=            -532.839920
 Sum of electronic and thermal Free Energies=         -532.881272
```

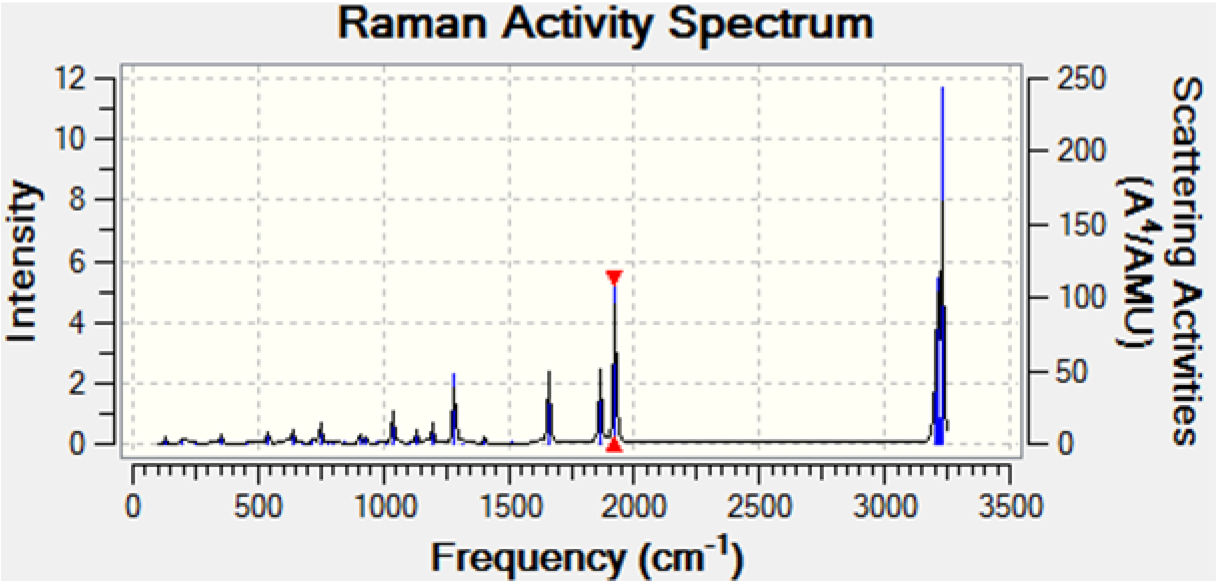

S46. Calculated Raman spectra of phthalic anhydride (B3LYP/6-31G*)

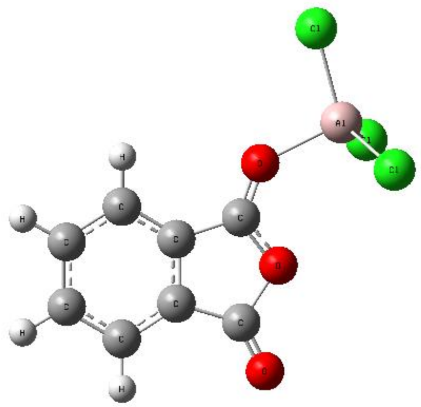

Phthalic anhydride AlCl3 complex B3LYP/6-31G*

0 1

C	4.65549200	1.07516100	-0.00018200
C	4.22001600	-0.25647300	-0.00008900
C	2.84829700	-0.46774100	0.00004600
C	1.94450300	0.59653900	0.00005300
C	2.36408200	1.92116000	-0.00006600
C	3.74597200	2.14336100	-0.00016700
H	5.72040500	1.28671300	-0.00026500
H	4.91909700	-1.08615500	-0.00011000
H	1.65179300	2.73960900	-0.00002200
H	4.12269100	3.16142900	-0.00023400
C	2.07219800	-1.72429200	0.00000200
O	2.37317800	-2.87222900	-0.00028300
C	0.60019500	0.01155900	-0.00002700
O	-0.46954200	0.62513900	0.00016500
O	0.67350000	-1.32373600	-0.00024300
Al	-2.34565000	0.11908600	0.00006300
Cl	-2.52213600	-0.98433900	1.81161900
Cl	-2.52296700	-0.98576400	-1.81045700
Cl	-3.26326000	2.03605900	-0.00085200

Zero-point correction= 0.109866
(Hartree/Particle)
Thermal correction to Energy= 0.124465
Thermal correction to Enthalpy= 0.125409
Thermal correction to Gibbs Free Energy= 0.064295
Sum of electronic and zero-point Energies= -2156.117619
Sum of electronic and thermal Energies= -2156.103020
Sum of electronic and thermal Enthalpies= -2156.102076
Sum of electronic and thermal Free Energies= -2156.163190

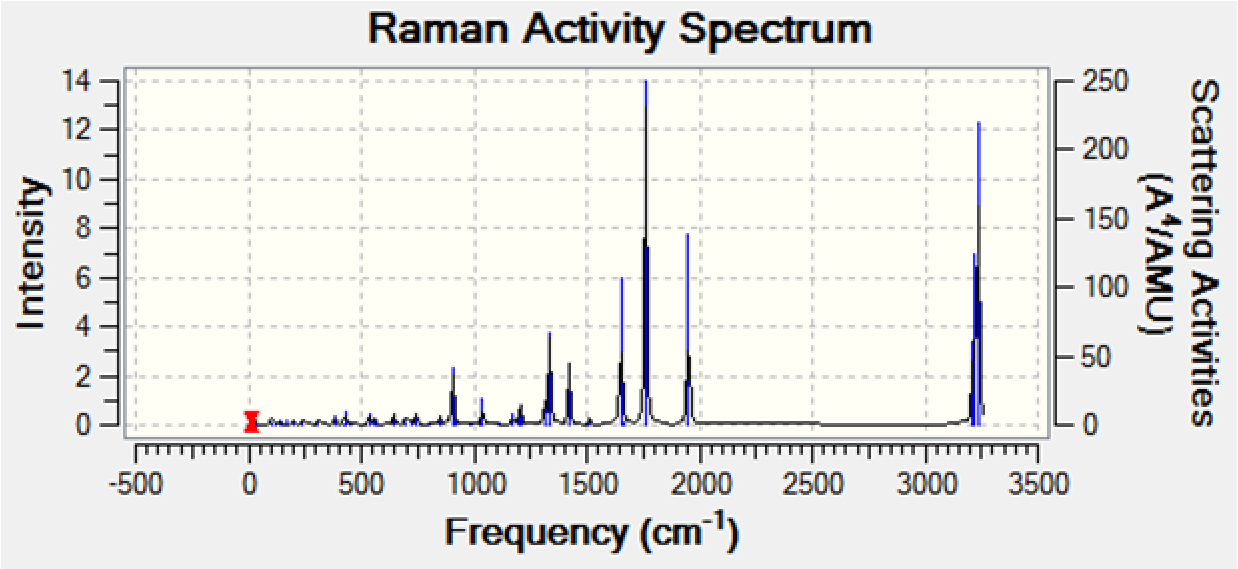

S47. Calculated Raman spectra of phthalic anhydride-$AlCl_3$ complex (B3LYP/6-31G*)

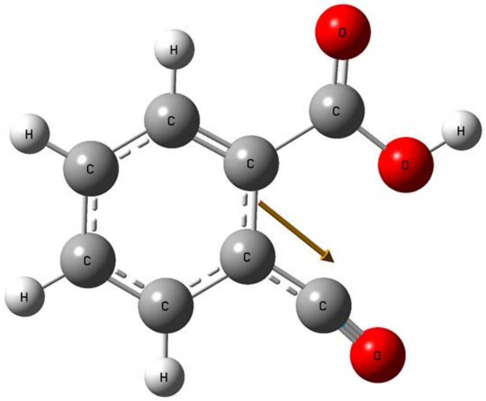

```
Acilium ion of phthalic anhydride B3LYP/6-31G*

1 1
 C                   2.37382600   -1.07394400    0.00009100
 C                   2.68358500    0.29132900    0.00003200
 C                   1.66667800    1.23700100   -0.00004100
 C                   0.32142100    0.78169800   -0.00003400
 C                   0.00008800   -0.60641200    0.00001800
 C                   1.04264700   -1.51828800    0.00008100
 H                   3.17685000   -1.80479200    0.00013600
 H                   3.71772300    0.61945600    0.00004300
 H                   1.89007800    2.29898300   -0.00008500
 H                   0.80262500   -2.57668300    0.00011600
 C                  -0.65729600    1.77430600   -0.00010400
 O                  -1.31179600    2.70354300   -0.00019600
 C                  -1.41304200   -1.10024200    0.00000500
 O                  -1.72872800   -2.25951600   -0.00045800
 O                  -2.27204800   -0.05273300    0.00053900
```

H   -3.19414300   -0.38000400   0.00041500

Zero-point correction=   0.113897
(Hartree/Particle)
Thermal correction to Energy=   0.122844
Thermal correction to Enthalpy=   0.123789
Thermal correction to Gibbs Free Energy=   0.079343
Sum of electronic and zero-point Energies=   -533.145273
Sum of electronic and thermal Energies=   -533.136326
Sum of electronic and thermal Enthalpies=   -533.135381
Sum of electronic and thermal Free Energies=   -533.179827

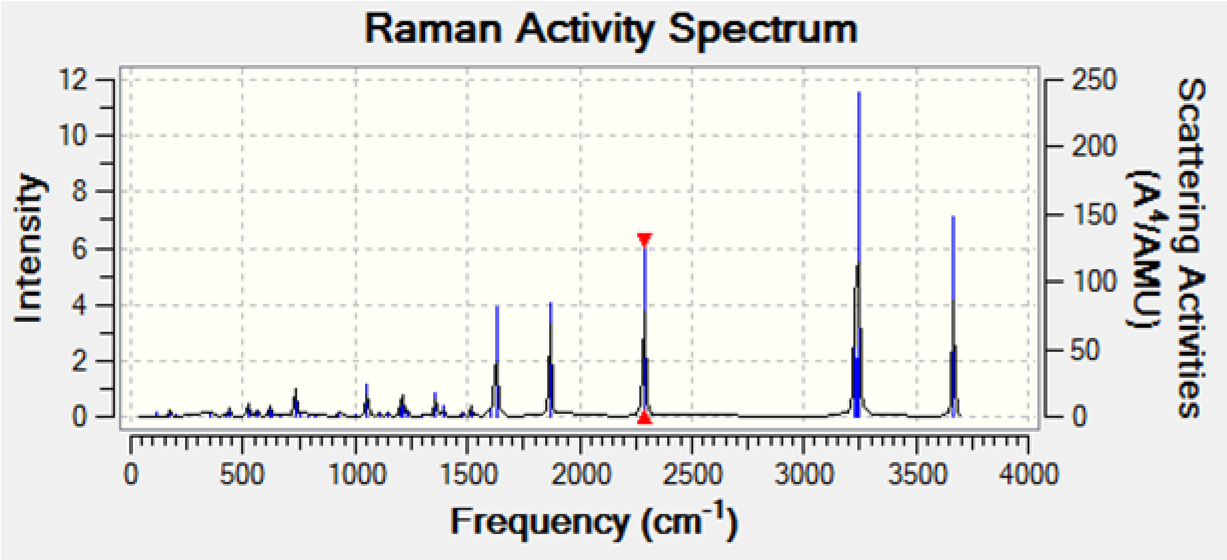

S48. Calculated Raman spectra of acilium ion of phthalic anhydride (B3LYP/6-31G*)

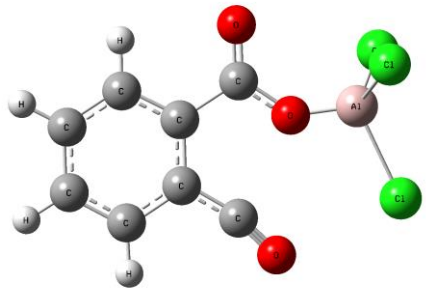

Acilium ion of phthalic anhydride AlCl3 complex B3LYP/6-31G*

Zero-point correction=   0.109054
(Hartree/Particle)
Thermal correction to Energy=   0.122270
Thermal correction to Enthalpy=   0.123214
Thermal correction to Gibbs Free Energy=   0.067462
Sum of electronic and zero-point Energies=   -2155.973537
Sum of electronic and thermal Energies=   -2155.960321

```
Sum of electronic and thermal Enthalpies=          -2155.959377
Sum of electronic and thermal Free Energies=       -2156.015129
```

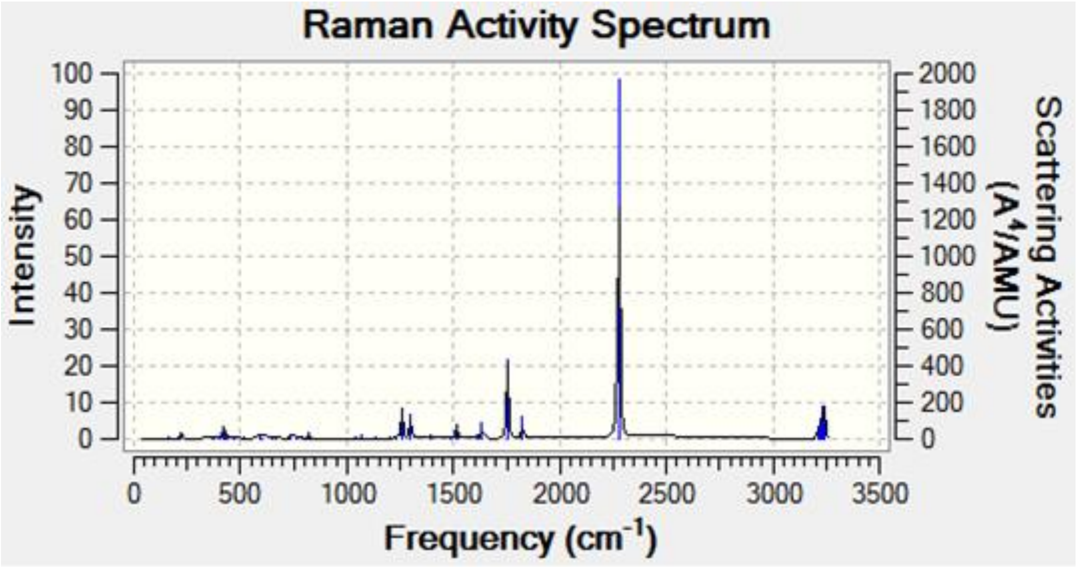

S49. Calculated Raman spectra of acilium ion of phthalic anhydride-$AlCl_3$ complex (B3LYP/6-31G*)

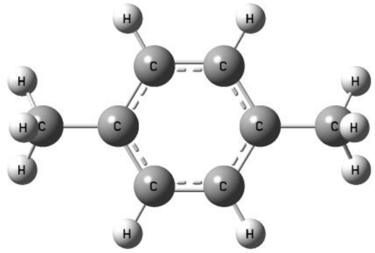

p-xylene B3LYP/6-31G*

```
0 1
 C        0.69739700    1.19847100   -0.01172600
 C       -1.42196900    0.00039100   -0.00779500
 C        1.42197400    0.00038200   -0.00740300
 H        1.23182300    2.14632900   -0.02129100
 C       -0.69718300   -1.19820000   -0.01186000
 C        0.69711500   -1.19824100   -0.01158900
 H       -1.23170500   -2.14602000   -0.02172300
 H        1.23157300   -2.14609500   -0.02127600
 C       -0.69735600    1.19847600   -0.01180500
 H       -1.23179100    2.14632000   -0.02135600
 C       -2.93300700   -0.00029700    0.02134800
 H       -3.34463300   -0.87188100   -0.49932700
 H       -3.31366900   -0.02935700    1.05145100
 H       -3.34406600    0.89905400   -0.44990300
 C        2.93303400   -0.00032900    0.02118000
```

```
 H                   3.34400800    0.89818700   -0.45175100
 H                   3.31401600   -0.02761800    1.05120900
 H                   3.34441400   -0.87283800   -0.49813100

Zero-point correction=                           0.155773
(Hartree/Particle)
 Thermal correction to Energy=                    0.163928
 Thermal correction to Enthalpy=                  0.164872
 Thermal correction to Gibbs Free Energy=         0.118778
 Sum of electronic and zero-point Energies=           -310.728734
 Sum of electronic and thermal Energies=              -310.720579
 Sum of electronic and thermal Enthalpies=            -310.719635
 Sum of electronic and thermal Free Energies=         -310.765729
```

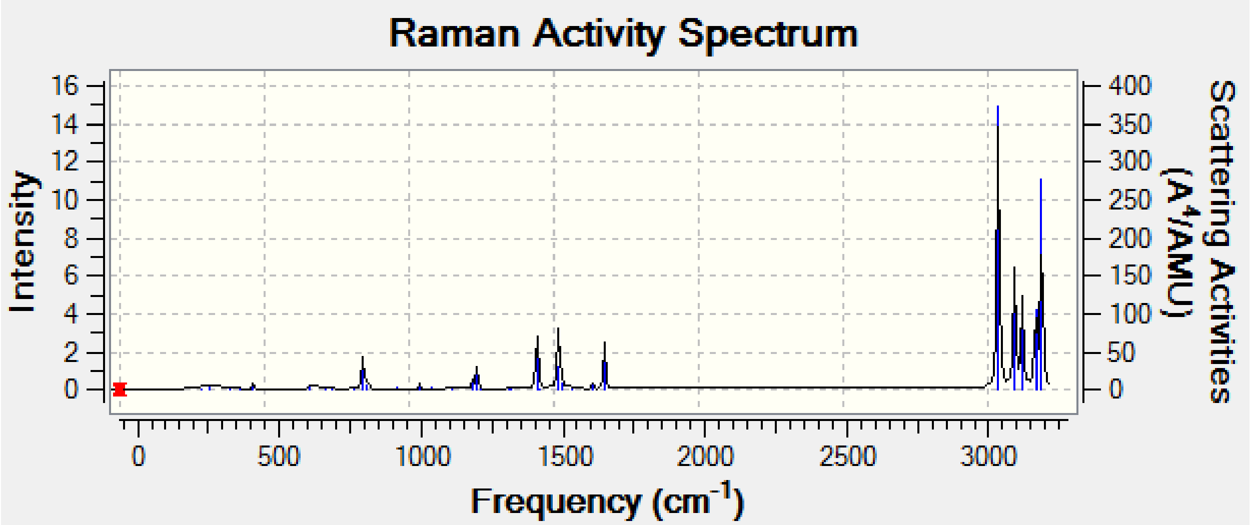

S50. Calculated Raman spectra of *p*-xylene (B3LYP/6-31G*)

References

1. https://cameochemicals.noaa.gov/chemical/8223
2. Casas-Solvas, J. M.; Mooibroek, T. J.; Sandramurthy, J D.; Howgego, J. D.; Davis, A. P. *Synlett* **2014**, *25*, 2591-2594.
3. Hahma, A.; Bhat, S.; Leivo, K.; Linnanto, J.; Lahtinen, M.; Rissanen, K. *New J. Chem*. **2008**, *32*, 1438-1448.
4. Zhang, Y.; He, B.; Liu, J.; Hu, S.; Pan, L.; Zhao, Z.; Tang, B. Z. *Phys. Chem. Chem. Phys.* **2018**, *20*, 9922-9929.
5. Zhang, T.-J.; Li, S.-Y.; Yuan, W.-Y.; Wu, Q.-X.; Wang, L.; Yang, S.; Sun, Q.; Meng, F.-H. *Bioorg. Med. Chem. Lett.* 2**017**, *27*, 729-732.
6. Jacob, B.; Sugunan, S.; Singh, A. P. *J. Mol. Catal. A* **1999**, *139*, 43-53.
7. Mohile, S. S.; Potdar, M. K.; Salunkhe M. M. *J. Chem. Research (S)* **2003**, 650-651.

NMR: Rosenfeld, S.; VanDyke, S. *J. Chem. Educ*. **1991**, *68*, 691-692.

8. Veerman, J.; van den Bergh, T.; Orrling, K. M.; Jansen, C.; Cos, P.; Maes, L.; Chatelain, E.; Ioset, J.-R.; Edink, E. E.; Tenor, H.; Seebeck, T.; de Esch, I.; Leurs, R.; Sterk, G. J. *Bioorg. Med. Chem.* **2016**, *24*, 1573-1581.
9. Parham W. E.; Bradsher, C. K.; Edgar. K. J. *J. Org. Chem.* **1981**, *46*, 1057-1061.
10. Zhang, T.-J.; Li, S.-Y.; Yuan, W.-Y.; Wu, Q.-X.; Wang, L.; Yang, S.; Sun, Q.; Meng, F.-H. *Bioorg. Med. Chem. Lett.* **2017**, *27*, 729-732.
11. Yamaguchi, M.; Kamei, K.; Koga, T.; Akima, M.; Kuroki, T.; Ohi, N. *J. Med. Chem.* **1993**, *36*, 4052-4060.
12. Dh. Hameed A. Dh.; Ovais, S.; Yaseen, R.; Rathore, P.; Samim, M.; Singh, S.; Sharma, K.; Akhtar, M.; Javed, K. *Arch. Pharm. Chem. Life Sci.* **2016**, *349*, 150-159.
13. Fujisawa, S.; Oonishi, I.; Aoki, J.; Ohashi, Y.; Sasada, Y. *Bull. Chem. Soc. Jpn.* **1985**, *58*, 3356-3359.
14. Mahmoodi, N. O.; Salehpour, M. *Russ. J. Org. Chem.* **2003**, *39*, 1760-1763.
15. Valters, R.; Bace, A.; Burkevica, A.; Kampare, R. *Zhur. Org. Khim.* **1976**, *12*, 173-7.
16. Serevičius, T.; Komskis, R.; Adoménas, P.; Adoménienė, O.; Jankauskas, V.; Gruodis, A.; Kazlauskas, K.; Juršėnas S. *Phys. Chem. Chem. Phys.* **2014**, *16*, 7089-7101.
17. Sartori, G.; Casnati, G.; Bigi, F.; Foglio, F. *Gazz. Chim. Ital.* **1990**, *120*, 13-19.
18. Sakai, N.; Kobayashi, T.; Ogiwara, Y. *Chem. Lett.* **2015**, *44*, 1503-1505.
19. Lu, G.; Franzén, R.; Zhang, Q.; Xu, Y. *Tetrahedron Lett.* **2005**, *46*, 4255-4259.
20. Breton, G. W.; Vang, X. *J. Chem. Educ.* **1998**, *75*, 81-82.
21. Alibert, S.; Santelli-Rouvier, C.; Castaing, M.; Berthelot, M.; Spengler, G.; Molnar, J.; Barbe, J. *Eur. J. Med. Chem.* **2003**, *38*, 253-263; Goh, Y. W.; White, J. M. *Aust. J. Chem.* **2009**, *62*, 419-424; Krivec, M.; Kranjc, K.; Polanc, S.; Kočevar, M. *Curr. Org. Chem.* **2011**, *18*, 1520-1527.
22. Margetić, D. *High Pressure Organic Synthesis*, de Gruyter, Berlin **2019**.
23. Margetić, D. Microwave Assisted Cycloaddition Reactions, Nova Science Publishers, Hauppauge **2011**.
24. Becke, A. D. *J. Chem. Phys.* **1993**, *98*, 5648-5652; Lee, C.; Yang, W.; Parr, R. G. *Phys. Rev. B* **1988**, *37*, 785-789.
25. GAUSSIAN 03, Revision B.03, M. J. Frisch, G. W. Trucks, H. B. Schlegel, G. E. Scuseria, M. A. Robb, J. R. Cheeseman, J. A. Montgomery, Jr., T. Vreven, K. N. Kudin, J. C. Burant, J. M. Millam, S. S. Iyengar, J. Tomasi, V. Barone, B. Mennucci, M. Cossi, G. Scalmani, N. Rega, G. A. Petersson, H. Nakatsuji, M. Hada, M. Ehara, K. Toyota, R. Fukuda, J. Hasegawa, M. Ishida, T. Nakajima, Y. Honda, O. Kitao, H. Nakai, M. Klene, X. Li, J. E. Knox, H. P. Hratchian, J. B. Cross, C. Adamo, J. Jaramillo, R. Gomperts, R. E. Stratmann, O. Yazyev, A. J. Austin, R. Cammi, C. Pomelli, J. W. Ochterski, P. Y. Ayala, K. Morokuma, G. A. Voth, P. Salvador, J. J. Dannenberg, V. G. Zakrzewski, S. Dapprich, A. D. Daniels, M. C. Strain, O. Farkas, D. K. Malick, A. D. Rabuck, K. Raghavachari, J. B. Foresman, J. V. Ortiz, Q. Cui, A. G. Baboul, S. Clifford, J. Cioslowski, B. B. Stefanov, G. Liu, A. Liashenko, P. Piskorz, I. Komaromi, R. L. Martin, D. J. Fox, T. Keith, M. A. Al-Laham, C. Y. Peng, A. Nanayakkara, M. Challacombe, P. M. W. Gill, B. Johnson, W. Chen, M. W. Wong, C. Gonzalez, and J. A. Pople, Gaussian Inc., Wallingford CT, 2004.
26. Scott, A. P.; Radom, L. *J. Phys. Chem.* **1996**, *100*, 16502-16513.
